# Supplementary material for: Paleontological and paleoecological significance of the oldest highly productive Upper Cretaceous (lowermost Maastrichtian) bonebed of Haţeg Basin (western Romania; Densuş-Ciula Formation)
Source: PLoS One. 2025 Nov 10;20(11):e0335893. doi: 10.1371/journal.pone.0335893 (PMC12599975; doi:10.1371/journal.pone.0335893)
Supplement: S1 File — (PDF) [file pone.0335893.s001.pdf]

Taphonomic file: model of taphonomic features recorded at K2 vertebrate site of Valiora locality (Haţeg Basin)

A – Serial number of bones

B – Inventory number of fossils

C, D, E – Maximum dimensions of bones

The three axes of most of the bones were measured where C was the largest dimension, D was the intermediate dimension, E was the smallest dimension.

F – Volume distribution of bones

The volume of bones was calculated from the measured data of the three axes of bones. The fossils were classified in the following categories based on the dataset of Pereda Suberbiola et al. (2000):

1, very small (<1cm^3)

2, small (1–16 cm^3)

3, medium (16–126 cm^3)

4, big (126–1000 cm^3)

5, very big (>1000 cm^3)

G – Maximum dimension of bones (Britt et al. 2009)

This category represents the original size of the bones in its pre-breakage state. We used the following size-class criteria:

1, very small <5 cm (maximum dimension of the whole or inferred whole element)

2, small 5 cm – 10 cm (maximum dimension of the whole or inferred whole element)

3, medium 10 cm – 50 cm (maximum dimension of the whole or inferred whole element)

4, big 50 cm – 100 cm (maximum dimension of the whole or inferred whole element)

5, very big >100 cm (maximum dimension of the whole or inferred whole element)

H – Number of symphysis

The presence or absence of epiphyses of limb bones was separately observed in this category:

1, both are present

2, only one is present

3, both of them are absent

I – State of the symphysis surface

1, intact

2, abraded

3, broken

4, broken during the preparation

5, wet rot

6, weathered before or after the fossilization

J – Abrasion

Bone abrasion refers to the alterations of the surface of bones and teeth, created by physical or chemical erosion before final burial (Behrensmeyer 1991):

1, unabraded

2, abraded

3, polished;

4, highly abraded (the internal surface of bone is exposed)

K – Weathering stage

Weathering is an indicator of the interaction of the temperature fluctuations, the moisture or other natural destructive effects acting on the bone surface (Behrensmeyer 1978):

1, unweathered

2, bone surface shows flaking associated with cracks

L – Breakage pattern

The breakage pattern provides information about whether the observed bone was fresh or already "fossilized" when it got broken. The following breakage types were distinguished where oblique (**2**), longitudinal (**3**) and (**4**) spiral are typical of the "pre-fossilization" breakages while the smooth transversal breakages (**5**) occur during the fossildiagenetic phase (Haynes 1983, Pereda Suberbiola et al. 2000, Ryan et al. 2001):

0, bone broken during the excavation or in the preparation phase

1, complete bone

2, oblique

3, longitudinal

4, spiral

5, smooth transversal

<u>M – Geometric form in pre-breakage state of bone:</u>

The shape of bones appears as an important factor in studying fluvial transport because the theoretical dispersal potential of fossils depends on the bone shape besides size and density (Behrensmeyer 1975, Frostic and Reid 1983):

1, spherical

2, rod-like

3, disc-shaped

4, other (blade-shaped, conical)

<u>N – crust:</u>

1, crust is absent on the bone surface

2, crust is present on the bone surface

<u>O – Deformation of bones by physical affect:</u>

1, the bone is flat and it is not deformed

2, not deformed

3, slightly deformed

4, highly deformed

<u>P – Ontogenetic stage</u>

1, juvenile

2, adult

3, hatchling

<u>Q – Anatomic position:</u>

It is an essential data for the MNI calculation:

1, right

2, left

R – Presence of teeth in their alveoli (in the premaxilla, maxilla or dentary)

1, more teeth are found in their alveoli

2, teeth are abraded in the maxilla or dentary

3, teeth are absent

S – Taxon:

Lepisosteus: **LEP**

Dinosauria: **DIN**

Zalmoxes: **ZA**

Telmatosaurus: **TE**

*Titanosauria***: TI**

Ornithopod?**: ORN**

Testudines: **T**

Allodaposuchus (?): **A**

Acynodon**: ACY**

*Theriosuchus***:THR**

*Doratodon***:DR**

Crocodilles: **CRO**

Theropods: **TH**

Pterosaurs: **PT**

*Hatzegopteryx***: HAT**

Kallokibotion: KA

Dortokidea: DORT

Sauopods:**SA**

Nidophis:**NID**

Lizard**:LIZ**

Anura**:AN**

Albanerpeton**:AL**

Kogaionidae**:KAG**

Eggshell fragments: **EGG**

T – Anatomical classification

S, complete or nearly complete skull

SE, skull element

J, complete or nearly complete jaw

JE, mandible element

MAX, maxilla

V, vertebra or vertebra fragment

CV, cervical vertebra

DV, dorsal vertebra

CAV, caudal vertebra

CHV, chevron

SACV, sacralis vertebrate

R, rib

COR, coracoid

SC, scapula

SAC, sacrum

PU, pubis

IL, ilium

ISC, ischium

ACE, acetabulum

PE, pelvis

HU, humerus

RA, radius

FI, fibula

U, ulna

TI, tibia

FE, femur

ME, metapodium

MEC, metacarpal

MET, metatarsus

PH, phalanx

HO, horn

CLA, claw

LIM, limb

PL, turtle plate

C, turtle carapax

P, turtle plastron

OST, osteoderm

SCU, fish scute

T, tooth

FRAG, fragment (unidentified bone)

TEN, tendon

U– Other taphonomic features

1, trampling effect

2, root track

3, tooth marks

4, insect boring

5, fungi marks

6, tectonically deformed

V – skeletalization

1, isolated

2, Associated

3, Articulated

W – Year
