# Supplementary material for: Paleontological and paleoecological significance of the oldest highly productive Upper Cretaceous (lowermost Maastrichtian) bonebed of Haţeg Basin (western Romania; Densuş-Ciula Formation)
Source: PLoS One. 2025 Nov 10;20(11):e0335893. doi: 10.1371/journal.pone.0335893 (PMC12599975; doi:10.1371/journal.pone.0335893)

Supplementary file:

BOTFALVAI ET AL:

Paleontological and paleoecological significance of the oldest highly productive Upper Cretaceous (lowermost Maastrichtian) bonebed of Haţeg Basin (western Romania; Densuş-Ciula Formation)

S3 File:

List and photos of the most important vertebrate taxa and specimens mentioned in the text.

Palynological assemblage collected from the site K2

Note:

See detailed in 4.2 subchapter and Figures 4-5 of the manuscript

Taxa	Samples (Fig 3B)		Figures
	6B	P2	
**Pteridophyta**			
Appendicisporites tricornitatus Weyland and Greifeld, 1953		1	
Biretisporites potoniaei Delcourt and Sprumont, 1955		2	
Cicatricosisporites curvatus Pu and Wu, 1982		3	
Cicatricosisporites dorogensis Potonie and Gelletich, 1933		4	
Cicatricosisporites sp.	4	3	
Deltoidospora minor (Couper, 1953) Pocock, 1970	7	5	
Deltoidospora hallii Miner, 1935		1	
Deltoidospora australis Pocock, 1970	7	2	Fig.4A
Deltoidospora punctatus (Delcourt and Sprumont, 1955) Delcourt et al., 1963		1	
Deltoidospora toralis (Leschik, 1955) Lund, 1977	1		
Deltoidospora sp.	9	2	
Dictyophyllidites sp		1	
Echinatisporis sp.	3		Fig.4H
Foveosporites sp.	1		
Gleicheniidites senonicus Ross, 1949	2	1	
Gleicheniidites sp.	2		
Laevigatosporites major (Cookson, 1947) Krutzsch, 1959		2	
Laevigatosporites ovatus Wilson and Webster, 1946	1	10	Fig.4F
Laevigatosporites sp.	2	4	
Leiotriletes sp.	1		
Lusatisporites dettmanniae (Drugg) Srivastava, 1972	4		Fig.4G
Lycopodiumsporites austroclavatidites (Cookson, 1953) Potonié, 1956	1		
Lycopodiumsporites clavatoides Couper, 1958		3	
Murospora sp.	1		
Osmundacidites sp.	3		
Polypodiaceoisporites hojrupensis Kedves, 1980	1		Fig.4E
Polypodiaceoisporites verruspeciosus Krutzsch, 1959		6	
Polypodiaceoisporites sp.	25	23	Fig.4B-D
Polypodiidites secundus (Potonié, 1934) Krutzsch, 1963	1		
Punctatisporites sp.	1		
Triplanosporites microsinuosus Pflanzl, 1955	1	5	
Triplanosporites sp.		2	
Verrucatosporites sp.	1		
Indeterminable spores	12	9	
**Bryophyta**			
Stereisporites antiquasporites (Wilson & Webster, 1946) Dettmann, 1963	1		Fig.4I
Stereisporites sp.		1	
**Gymnospermatophyta**			
Araucariacites australis Cookson, 1947	4	1	Fig.4M
Araucariacites sp.		1	
Cedripites sp.	1		
Classopollis classoides (Pflug, 1953) Pocock and Jansonius, 1961	1		
Classopollis sp.	8	1	Fig.4L
Cycadopites sp.		2	
Inaperturopollenites dubius (Potonié & Venitz, 1934) Thomson & Pflug, 1953	1		Fig.4J
Pinuspollenites sp.	5		Fig.4K
**Angiospermatophyta**			
Clavatipollenites sp.	1		
Fraxinoipollenites sp.		1	
Myricipites bituitus (Potonié, 1931) Nagy, 1969	1		Fig.4T
Myricipites sp.	4		
Oculopollis praedicatus (Weyland and Krieger, 1953) emend. Polette and Batten, 2017		1	Fig.4N
Plicapollis serta Pflug, 1953	2	4	
Proteacidites sp.	4		
Pseudopapillopollis praesubhercynicus (Góczán, 1964) Góczán et al., 1967	1		
Subtriporopollenites anulatus Pflug and Thomson, 1953		1	Fig.4U
Subtriporopollenites constans constans (Pflug, 1953) Kedves, 1974	2		
Subtriporopollenites sp.	1		
Tricolpites sp.	3		
Trudopollis minimus Góczán, 1964	3		Fig.4S
Trudopollis nonperfectus (Pflug in Thomson and Pflug, 1953) Pflug, 1953		2	
Trudopollis cf. *primigenius* Krutzsch, 1973		1	
Trudopollis sp.		1	
Triatriopollenites sp.		4	
Indeterminable Normapolles		2	
**Freshwater algae**			
Chomotriletes fragilis Pocock, 1962		2	Fig.4V
Ovoidites sp.	1		Fig.4X
**Reworked palynomorphs (dinoflagellate cysts and spores)**			
Impagidinium sp.	10		
Pterodinium cingulatum cf. *granulatum* (Clarke and Verdier, 1967) Lentin and Williams, 1981	17		Fig.4Y-Z
indeterminable dinocysts	3		
spores	16		

Molluscs assemblage collected from the site K2

Note:

See detailed in 4.3 subchapter and Figures 6-7 of the manuscript

Taxa	Samples (Fig 3B)		Figures
	Flood bed 1	Flood bed 1	
Physidae indet.	110	6	
Physidae sp1			Fig.6C
Physidae sp2			Fig.6D
Physidae sp3			Fig.6E
Physidae sp4			Fig.6F-H
Physidae sp5			Fig.6I-J
Lymnaeidae indet. (or terrestrial gastropod)	33		Fig.6A
Anostomopsidae indet	3		
Cyclophoridae indet	1		
Helicoidea indet	11		
Helicoidea sp1			Fig.7C-D
Helicoidea sp2			Fig.7E
Helicoidea sp3			Fig.7F-G
Ajkaia sp.	4		Fig.6K
Pupinidae indet	15	4	
Pupinidae sp1			Fig.6l-N
Pupinidae sp2			Fig.6O-Q
Bithynia sp.	2		
Acroloxus sp.	1		Fig.6B
High-spired gastropod	3		
Lychnus sp.	1		Fig.7A-B
"Ribbed" Pupinidae indet.	1		
Laminiferinae indet.	1		
Sphaeriidae indet. (and one of these maybe Cyrenidae)	270		
Sphaeriidae sp1			Fig.7L
Sphaeriidae sp2			fig.7M
Sphaeriidae sp3			Fig.7N
Sphaeriidae sp4			Fig.7O
dextral slender	1		

Vertebrate assemblage collected from the site K2

Note:

See detailed in 4.4 subchapter and Figures 8-11 of the manuscript

Taxa	anatomy	Samples (Fig 3B)		Figures	Inventory number	References
		Flood bed 1	Flood bed 2			
**Isolated vertebrate matarial**						
Lepisosteiformes indet.	scale	X		Fig.8A	v.900	this study
Albanerpetontidae indet.	dentary	X		Fig8B	v.901	this study
Anura indet	limb bones	X				this study
Barbatteidae indet.	maxilla	X		Fig8C	v.902	this study
Kallokibotion sp.	complete shell	X			R.2710	Botfalvai et al 2021
Doratodon sp.	tooth	X		Fig8G	V907	this study
Doratodon sp.	tooth	X			V908	this study
Doratodon sp.	tooth	X			V909	this study
Doratodon sp.	tooth	X			V910	this study
Acynodon sp.	tooth	X		Fig8J	V903	this study
Acynodon sp.	tooth	X			V904	this study
Acynodon sp.	tooth	X			V905	this study
Acynodon sp.	tooth	X			V906	this study
Acynodon sp.	tooth	X			V933	this study
Theriosuchus -like sp.	tooth	X		Fig8H	V915	this study
Theriosuchus -like sp.	tooth	X			V916	this study
Theriosuchus -like sp.	tooth	X			V917	this study
Theriosuchus -like sp.	tooth	X			V918	this study
Theriosuchus -like sp.	tooth	X			V919	this study
Theriosuchus -like sp.	tooth	X			V923	this study
Theriosuchus -like sp.	tooth	X			V924	this study
Theriosuchus -like sp.	tooth	X			V925	this study
Theriosuchus -like sp.	tooth	X			V926	this study
Theriosuchus -like sp.	tooth	X			V927	this study
Theriosuchus -like sp.	tooth	X			V928	this study
Theriosuchus -like sp.	tooth	X			V929	this study
Theriosuchus -like sp.	tooth	X			V930	this study
Theriosuchus -like sp.	tooth	X			V931	this study
Theriosuchus -like sp.	tooth	X			V932	this study
Allodaposuchus sp.	tooth	X		Fig8I	V911	this study
Allodaposuchus sp.	tooth	X			V912	this study
Allodaposuchus sp.	tooth	X			V913	this study
Allodaposuchus sp.	tooth	X			V914	this study
Crocodiliformes indet.	tooth	X			V920	this study
Crocodiliformes indet.	tooth	X			V921	this study
Crocodiliformes indet.	tooth	X			V922	this study
Hadrosairia indet.	Tooth	X				this study
Hadrosairia indet.	Tooth	X				this study
Hadrosairia indet.	Dentary fragment	X		Fig. 8P	R.2887	this study
Richardoestesia sp.	tooth	X		Fig. 8N-O	R.2884	this study
Richardoestesia sp.	tooth	X			R.2886	this study
Velociraptorine dromaeosaurid	tooth	X		Fig. 8L-M	R.2885	this study
Theropoda indet.	Limb bone fragments	X				this study
Theropoda indet.	Limb bone fragments	X				this study
Theropoda indet.	Limb bone fragments	X				this study
Theropoda indet.	Caudal vertebra	X				this study
Pterosauria indet.	Limb bone fragments	X		Fig8Q	R.2891	this study
Multituberculata indet.	tooth	X		Fig. 8R-S	M.1710	this study

Taxa	anatomy	Samples (Fig 3B)		Figures	Inventory number	References
		Flood bed 1	Flood bed 2			
**Assiciated and articulated Rhabdodontidae indet. material**						
Rhabdodontidae indet.	premaxilla	X		Fig10A	R.2769	Magyar et al 2024
Rhabdodontidae indet.	maxilla	X		Fig10B	R.2770	Magyar et al 2024
Rhabdodontidae indet.	prefrontal	X		Fig10C	R.2772	Magyar et al 2024
Rhabdodontidae indet.	frontal-postorbital	X		Fig10D	R.2774	Magyar et al 2024
Rhabdodontidae indet.	dentary	X		Fig10E	R.2778	Magyar et al 2024
Rhabdodontidae indet.	vertebra	X		Fig10F	R.2795	Magyar et al 2024
Rhabdodontidae indet.	chevron	X		Fig10G	R.2805	Magyar et al 2024
Rhabdodontidae indet.	scapula	X		Fig10H	R.2806	Magyar et al 2024
Rhabdodontidae indet.	ulna	X		Fig10I	R.2807	Magyar et al 2024
Rhabdodontidae indet.	illium	X		Fig10J	R.2809	Magyar et al 2024
Rhabdodontidae indet.	ischium	X		Fig10K	R.2810	Magyar et al 2024
Rhabdodontidae indet.	tibia	X		Fig10L	R.2812	Magyar et al 2024
Rhabdodontidae indet.	fibula	X		Fig10M	R.2814	Magyar et al 2024
Rhabdodontidae indet.	metatarsal	X		Fig10N	R.2816	Magyar et al 2024
Rhabdodontidae indet.	phalanx	X		Fig10O	R.2822	Magyar et al 2024
**Assiciated and articulated Titanosauria indet. material**						
Titanosauria indet.	dorsal vertebra	X		Fig11A	R.2896	this study
Titanosauria indet.	sacral rib	X		Fig11B	R.2897	this study
Titanosauria indet.	caudal vertebra	X		Fig11C	R.2715	this study
Titanosauria indet.	caudal vertebra	X		Fig11D	R.2715	Botfalvai et al 2021
Titanosauria indet.	caudal vertebra	X		Fig11E	R.2898	this study
Titanosauria indet.	chevron	X		Fig11F	R.2899	this study
Titanosauria indet.	humerus	X		Fig11G	R.2900	this study
Titanosauria indet.	metapodium	X		FIg11H	R.2091	this study
Titanosauria indet.	pubis	X		Fig11I	R.2902	this study
Titanosauria indet.	Femur	X		Fig11J	R.2903	this study
Titanosauria indet.	Femur	X		Fig11K	R.2904	this study
Titanosauria indet.	Femur	X		Fig11L	R.2905	this study
Titanosauria indet.	Tibia	X		Fig11M	R.2906	this study
Titanosauria indet.	Fibula	X		Fig11N	R.2907	this study
Titanosauria indet.	phalanx	X		Fig11O	R.2908	this study

Larger images of the fossils described in detail in the manuscript. The specimens shown here are shown in Figures 8, 10, and 11.

Figure 8: Isolated vertebrate remains, site K2, Vălioara (Densuș–Ciula Formation).

<u>Note:</u> The scanning electron microscope photos of different specimens are not shown here because their resolution is sufficient in the Figure 8 published in the manuscript.

Richardoestesia sp. tooth, (LPB [FGGUB] R.2884) in labial view.

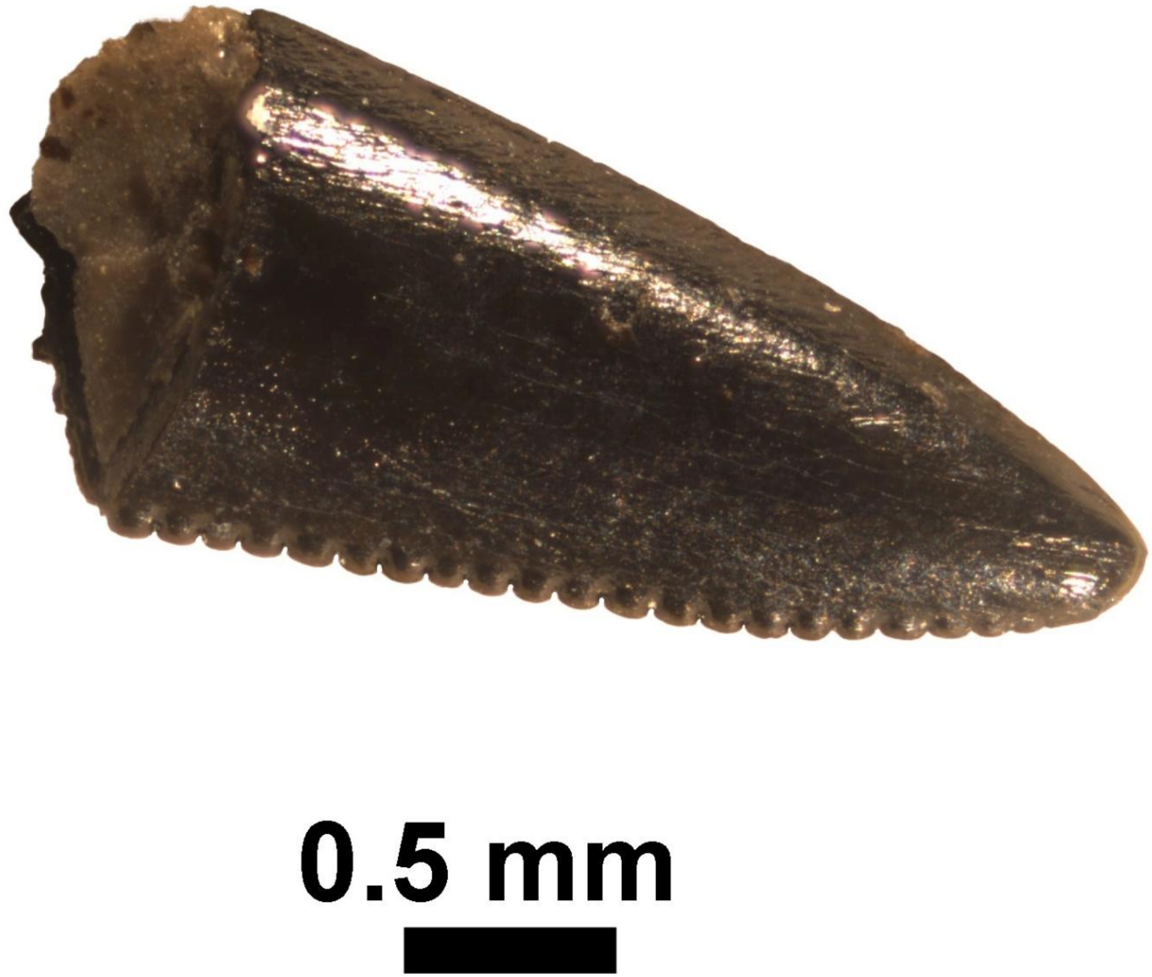

Richardoestesia sp. tooth, (LPB [FGGUB] R.2884) in lingual view.

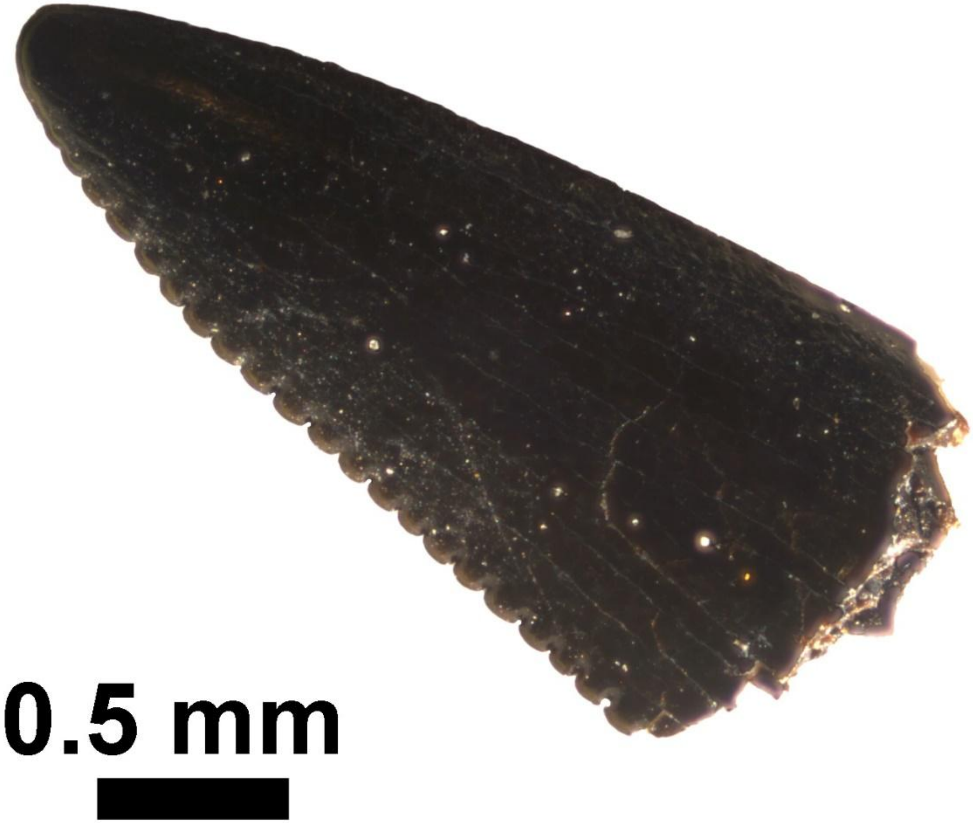

Velociraptorine dromaeosaurid theropod tooth, LPB [FGGUB] R.2885 in labial view.

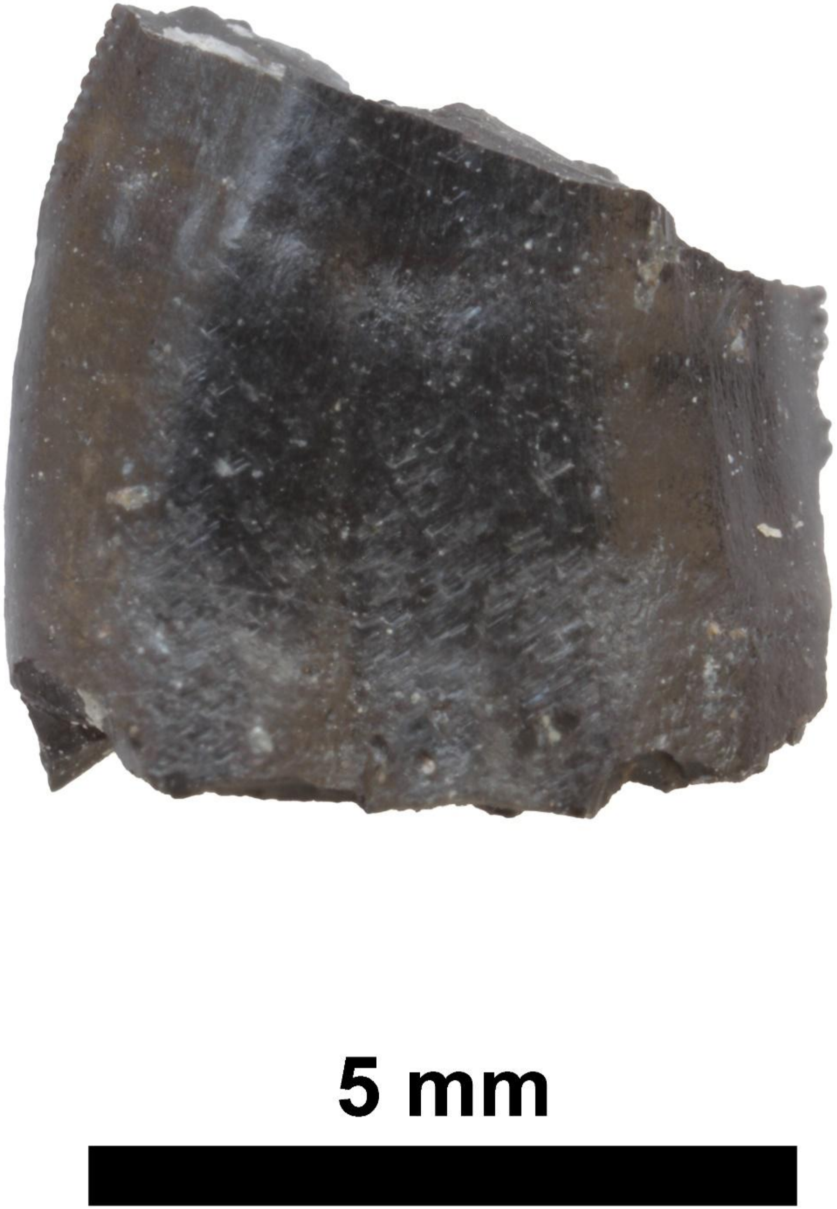

Velociraptorine dromaeosaurid theropod tooth, LPB [FGGUB] R.2885 in lingual view.

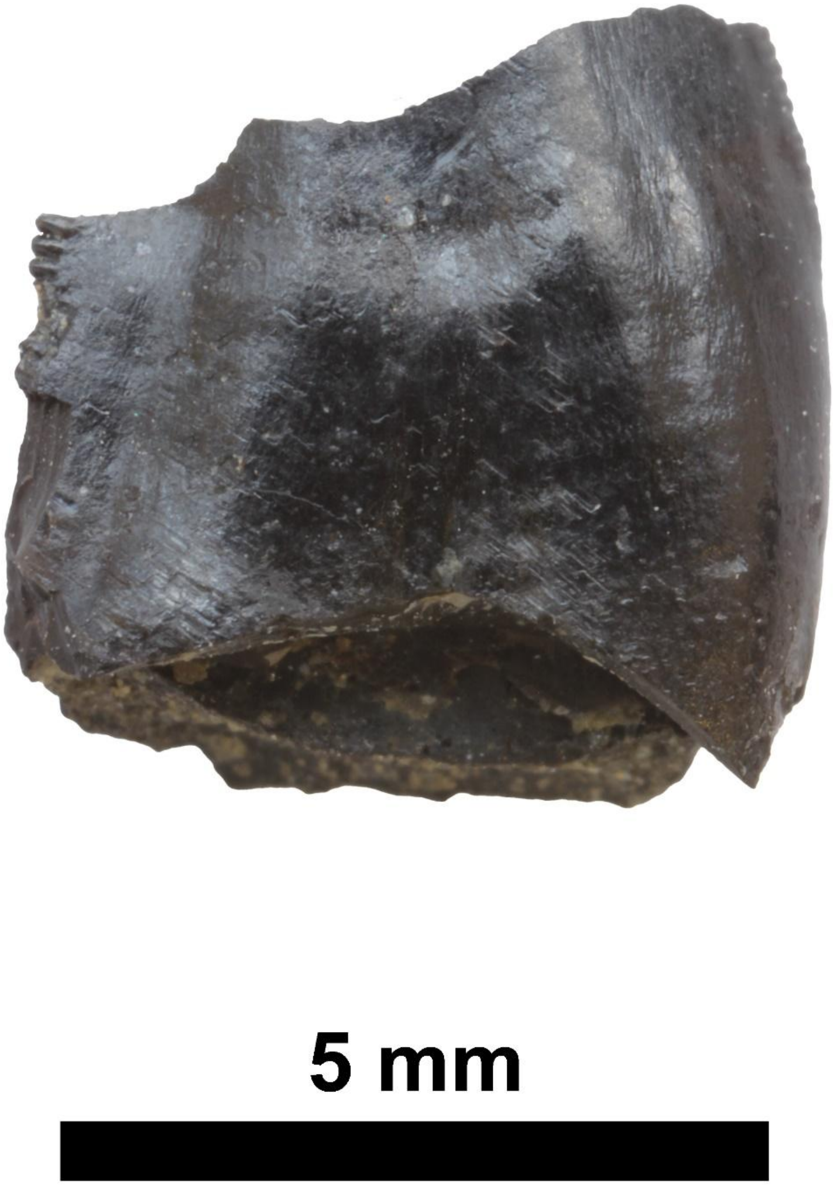

Hadrosauroidea indet., right dentary fragment LPB [FGGUB] R.2887 in medial view.

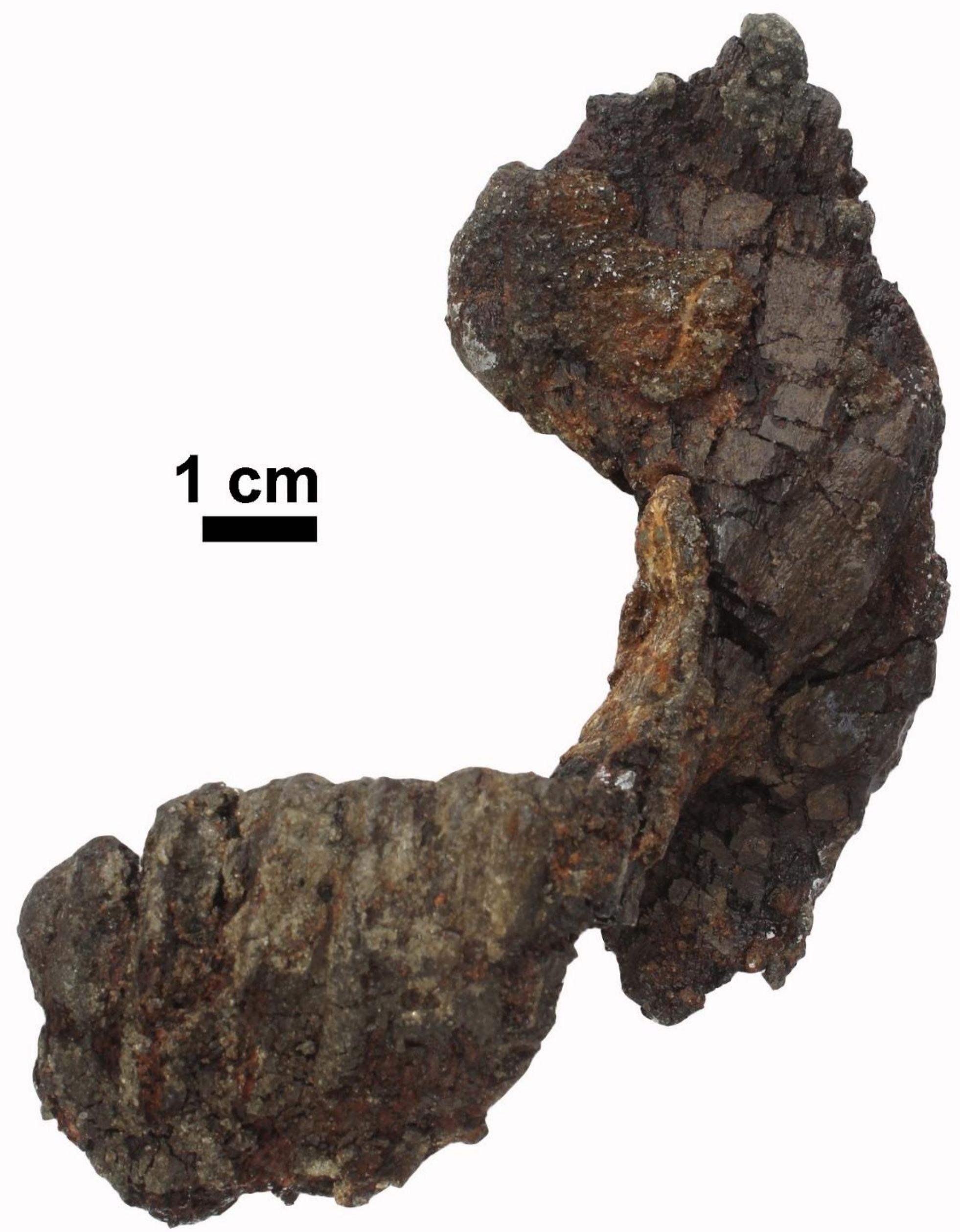

Possible pterosaurian limb bone fragment LPB [FGGUB] R.2891.

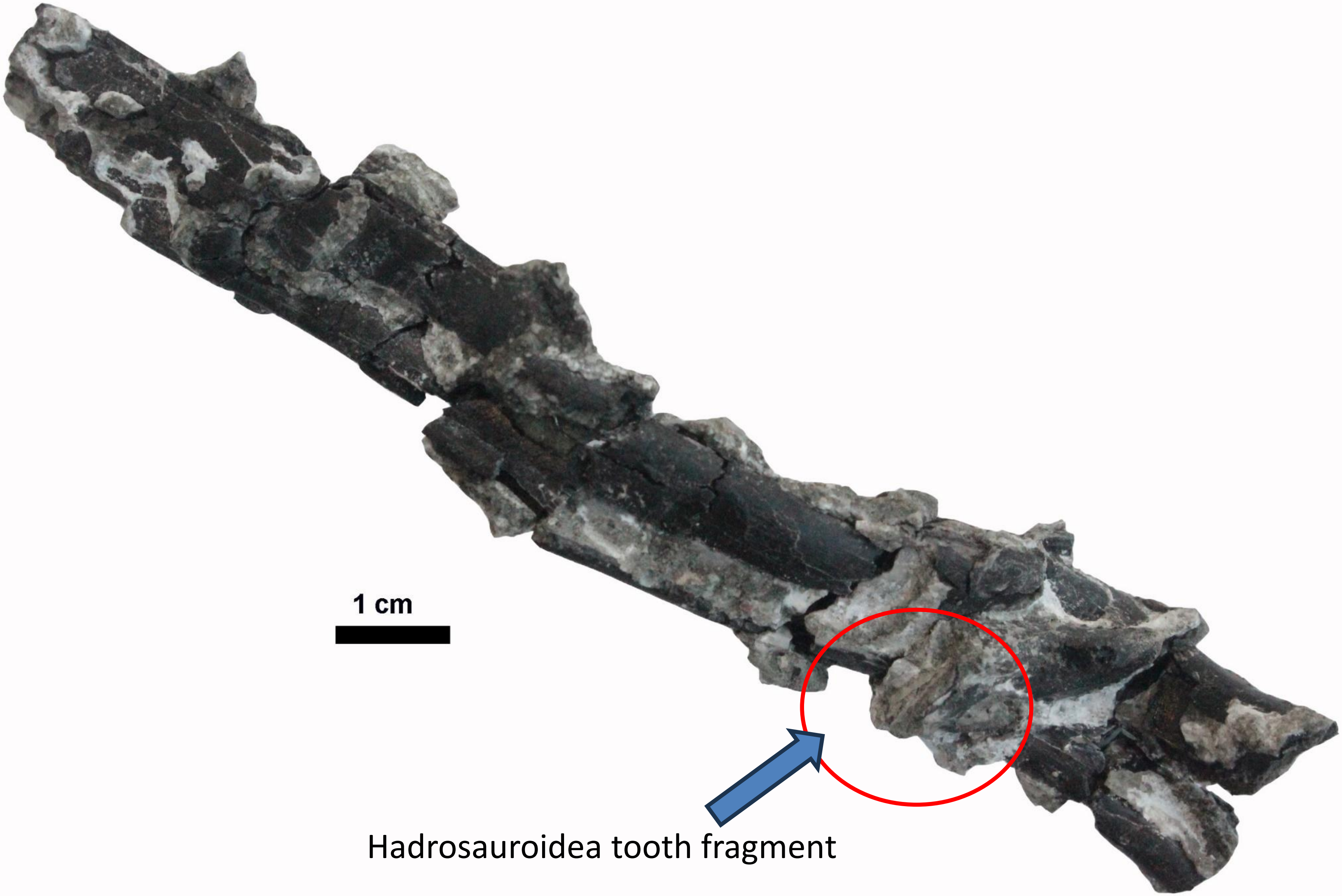

Figure 10: Generalized cranial and skeletal reconstruction of the 'K2 rhabdodontid', Vălioara (Densuș–Ciula Formation).

Note: The bone material presented here has already been published in Magyar et al 2024.

See:

https://www.sciencedirect.com/science/article/abs/pii/S0195667123003385

Associated premaxillae, LPB (FGGUB) R.2769 in left lateral view.

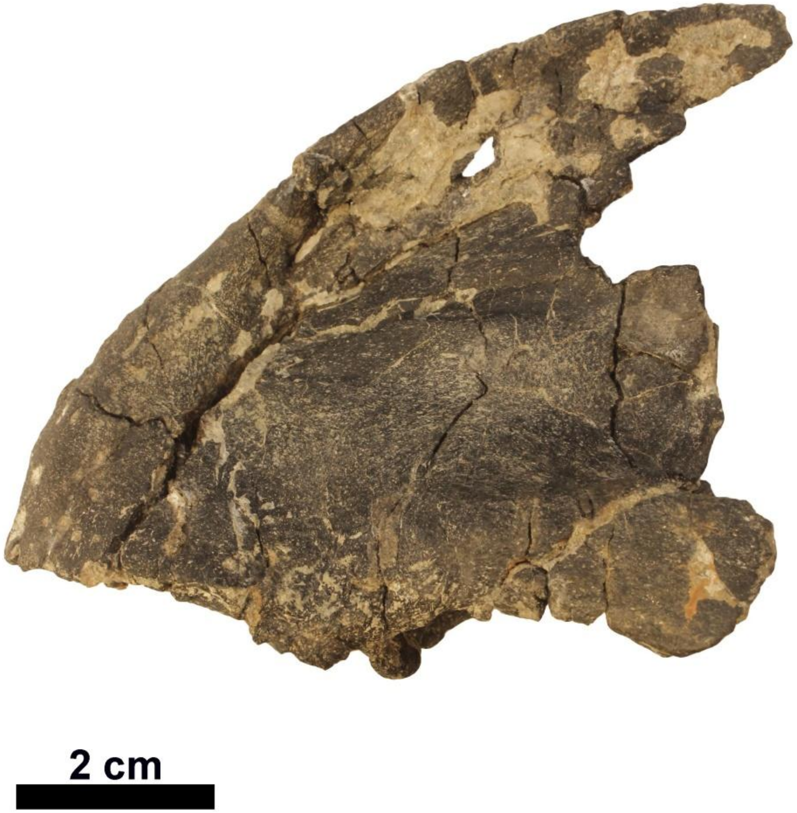

Right maxilla, LPB (FGGUB) R.2770, in lateral view.

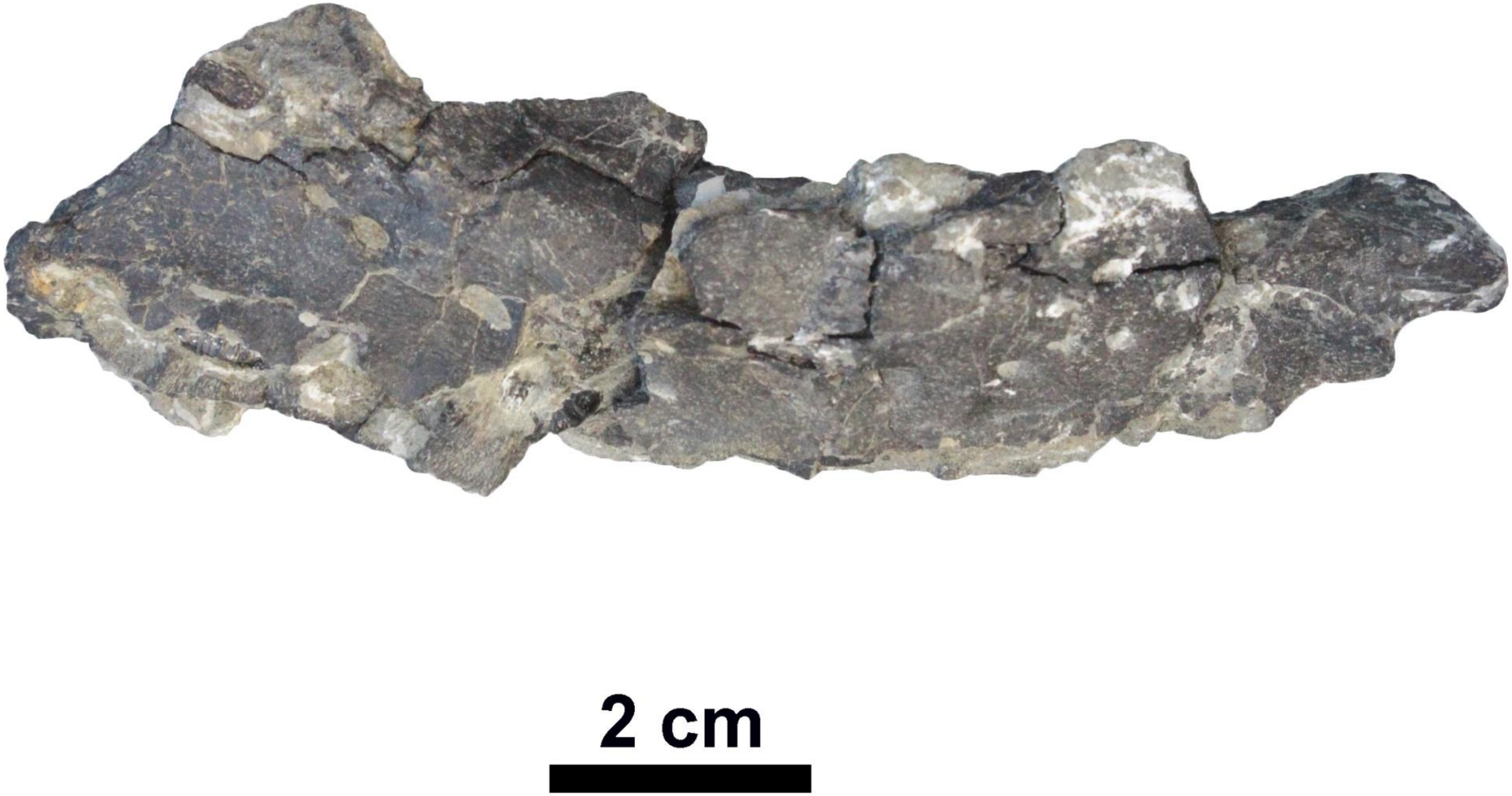

Left prefrontal, LPB (FGGUB) R.2772, in dorsal view.

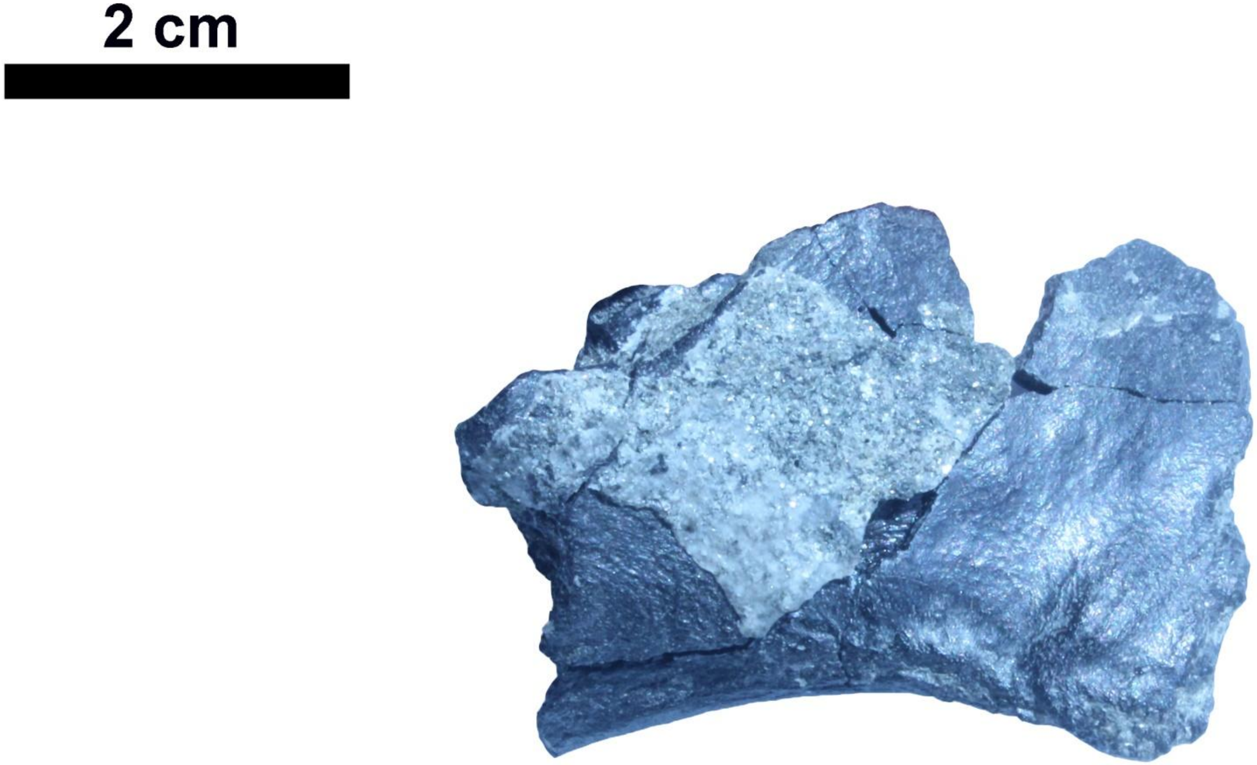

Articulated right frontal-postorbital, LPB (FGGUB) R.2774, in dorsal view.

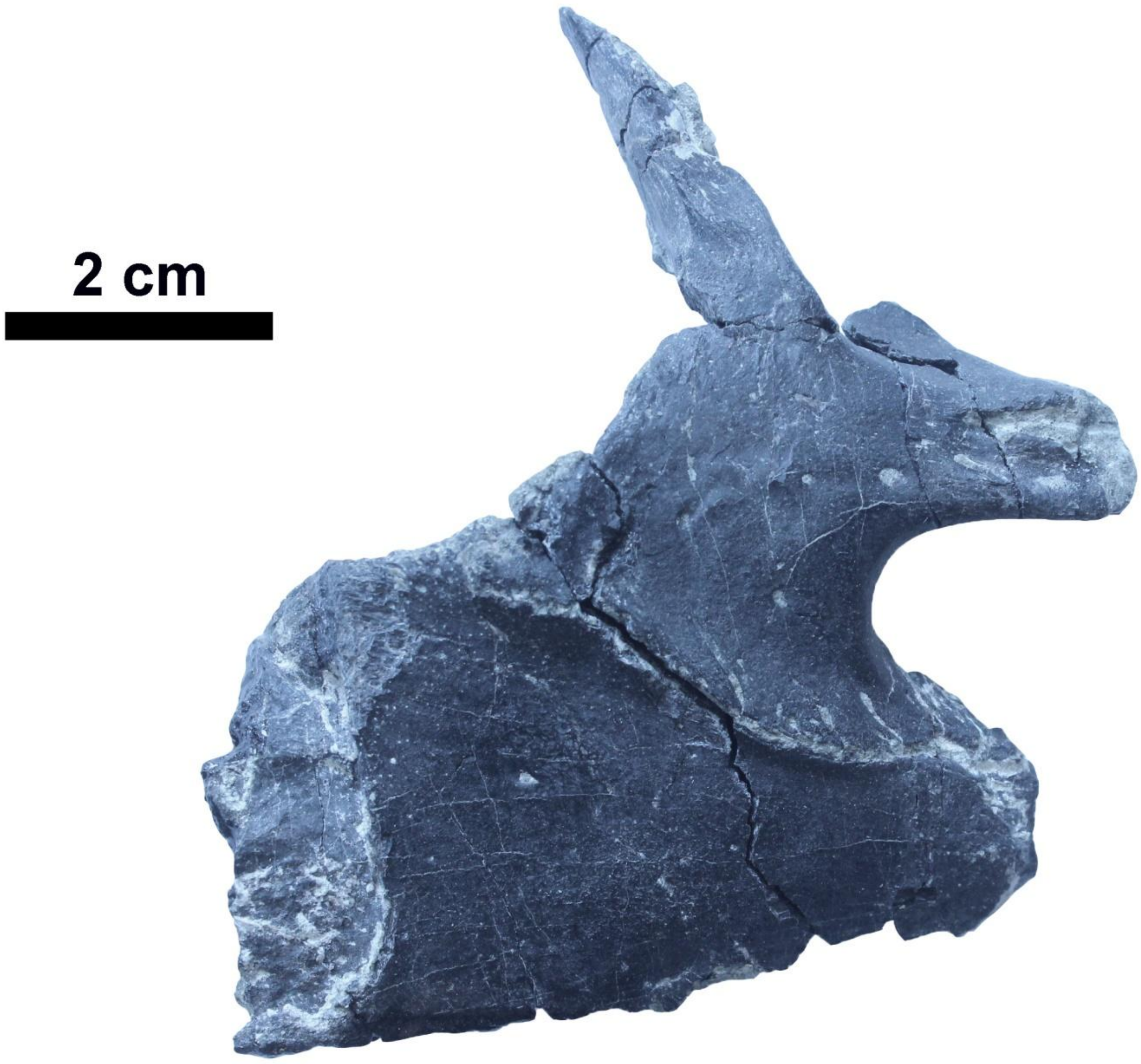

Right dentary, LPB (FGGUB) R.2778, in medial view.

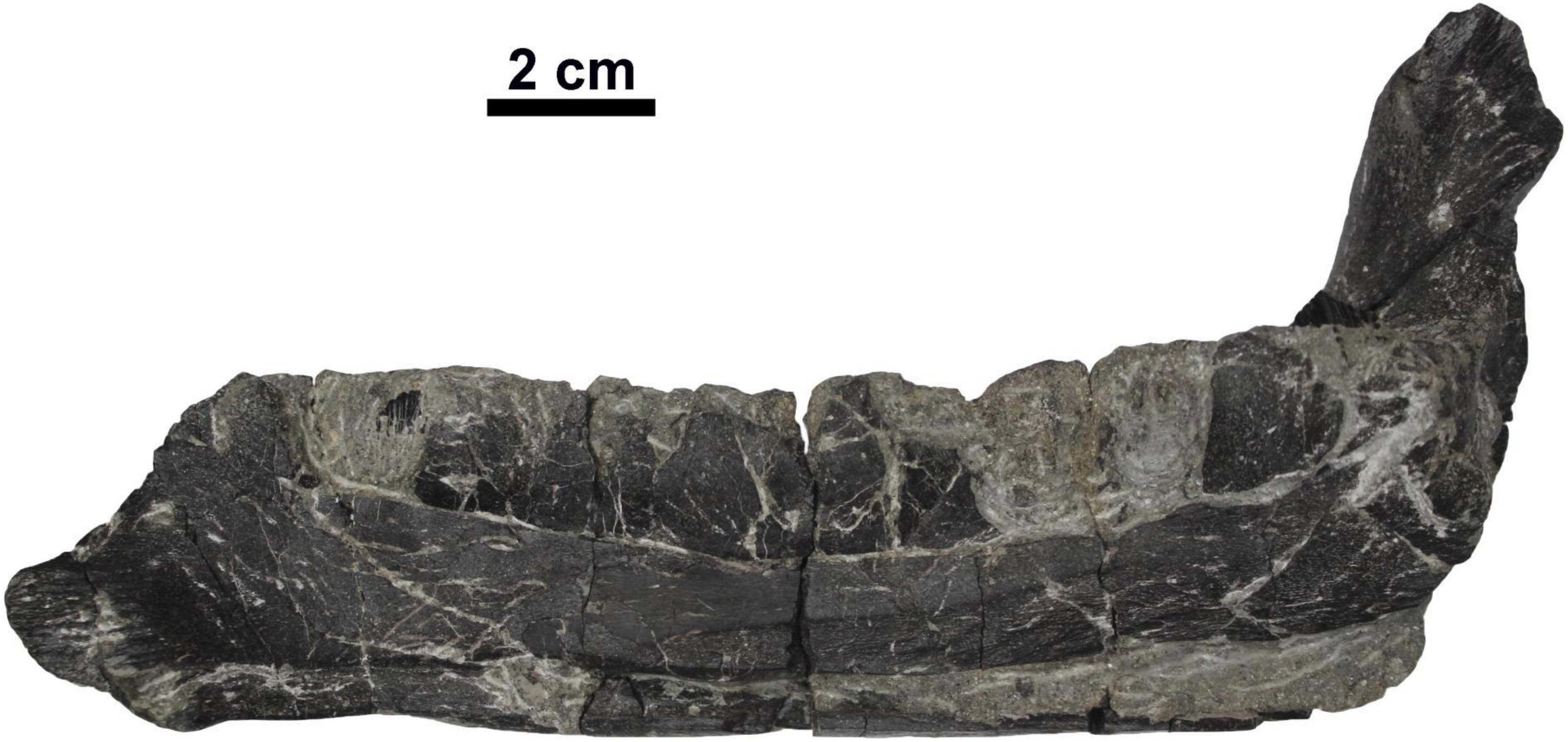

Middle dorsal vertebra, LPB (FGGUB) R.2795, in posterior view.

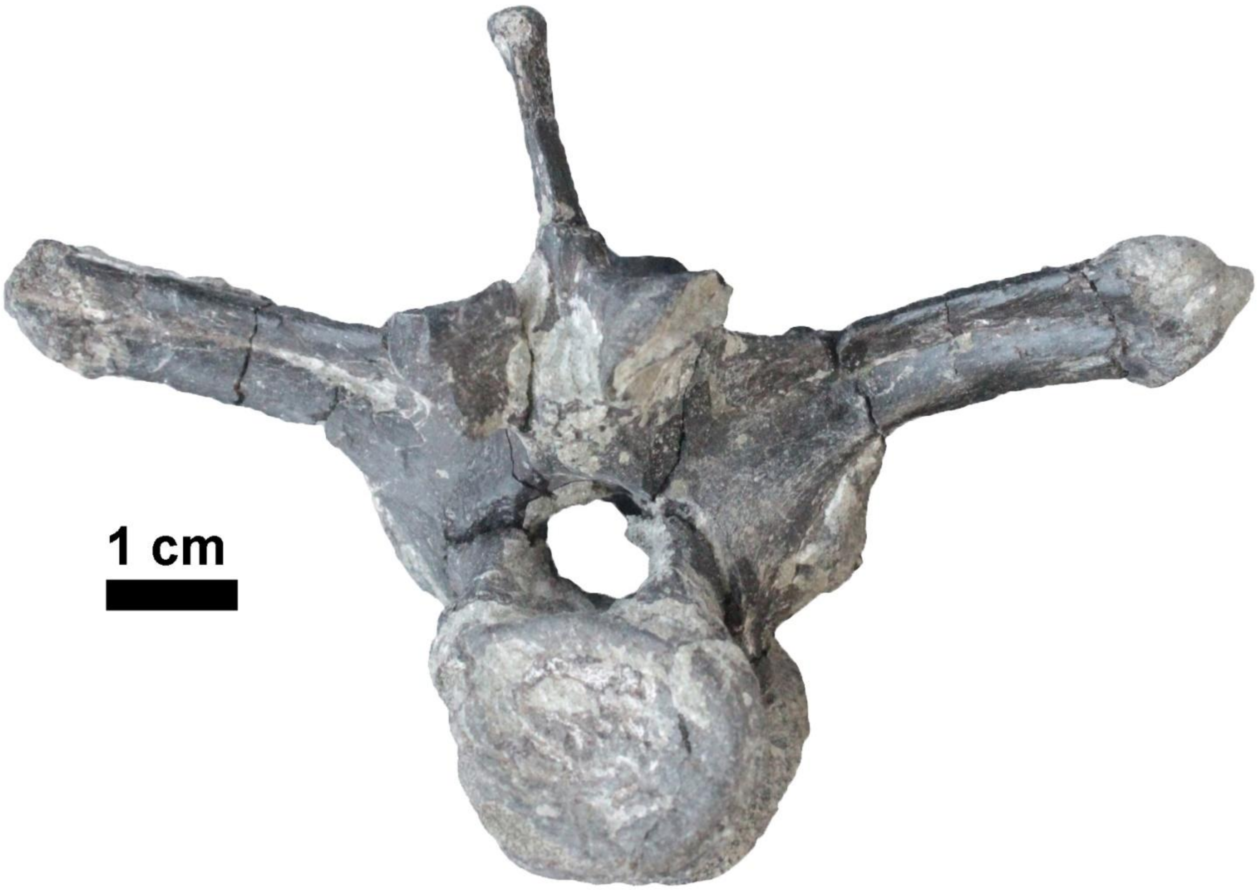

Chevron, LPB (FGGUB) R.2805, in anterior view.

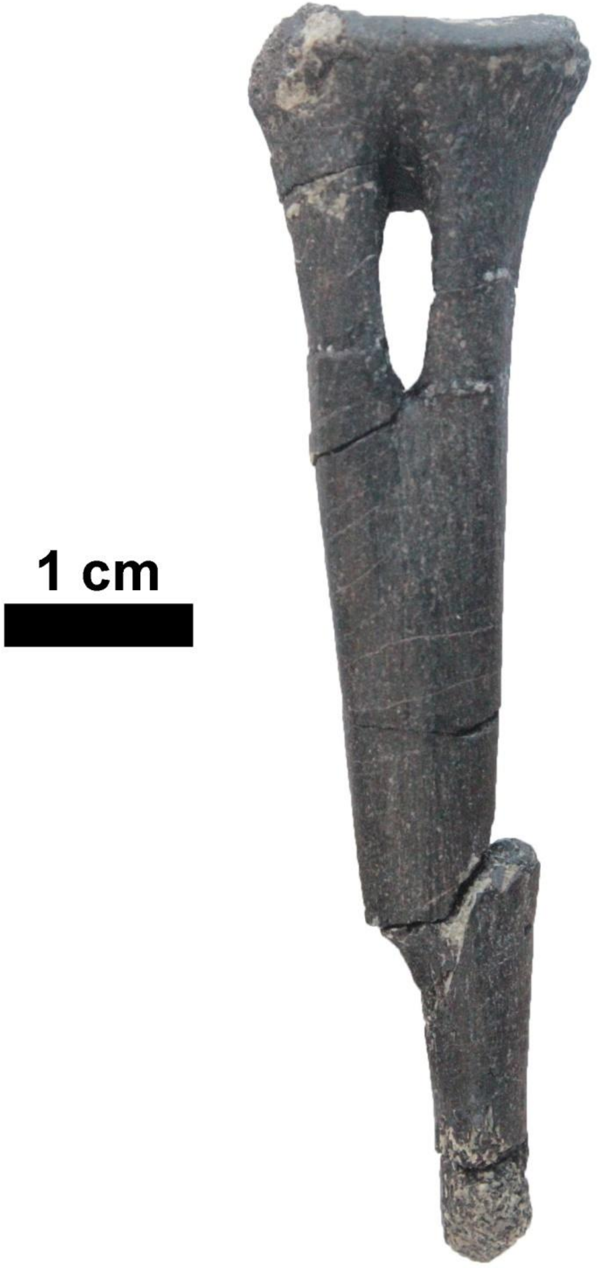

Right scapula, LPB (FGGUB) R.2806, in lateral view.

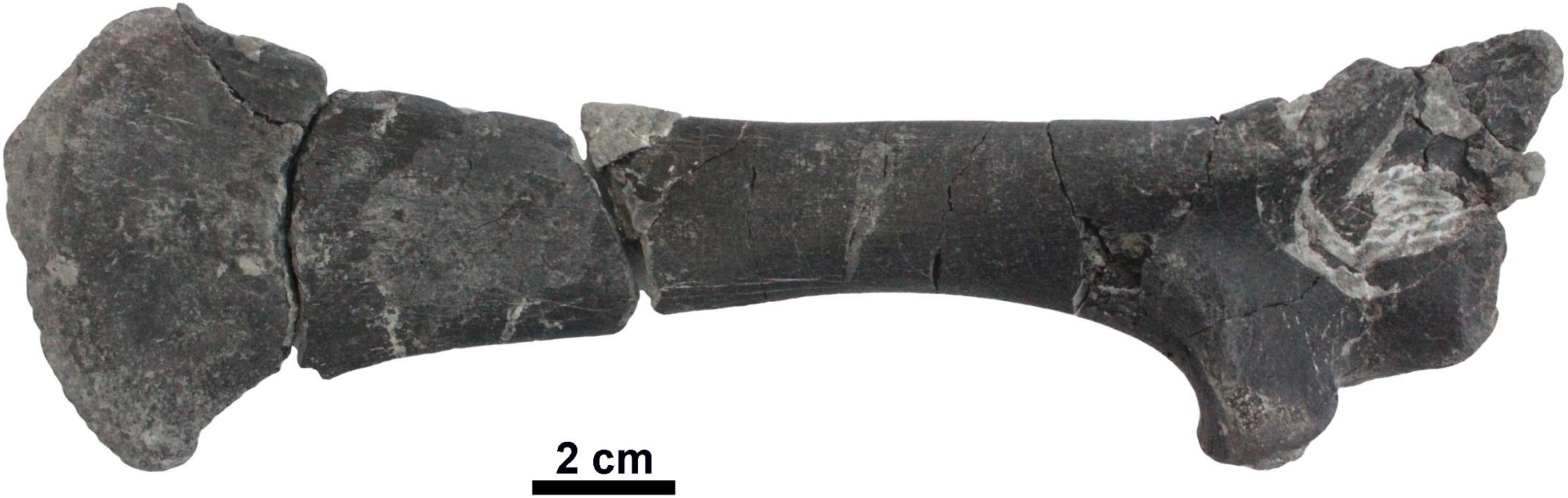

Right ilium, LPB (FGGUB) R.2809, in lateral view.

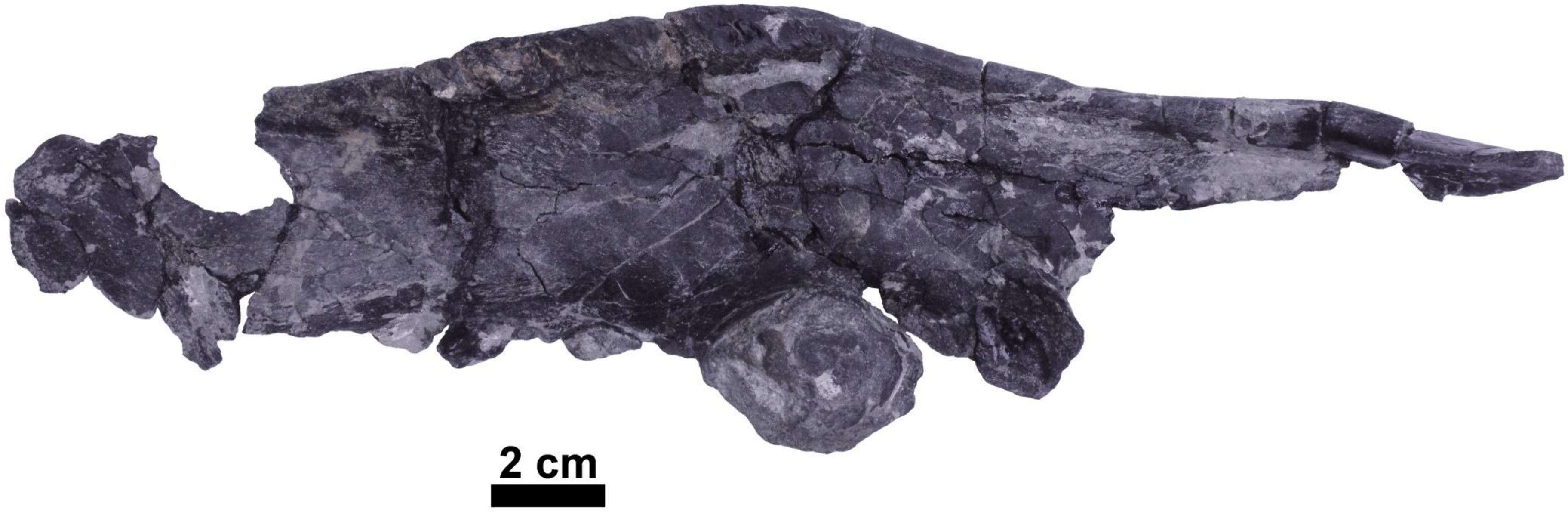

Left ischium, LPB (FGGUB) R.2810, in lateral view.

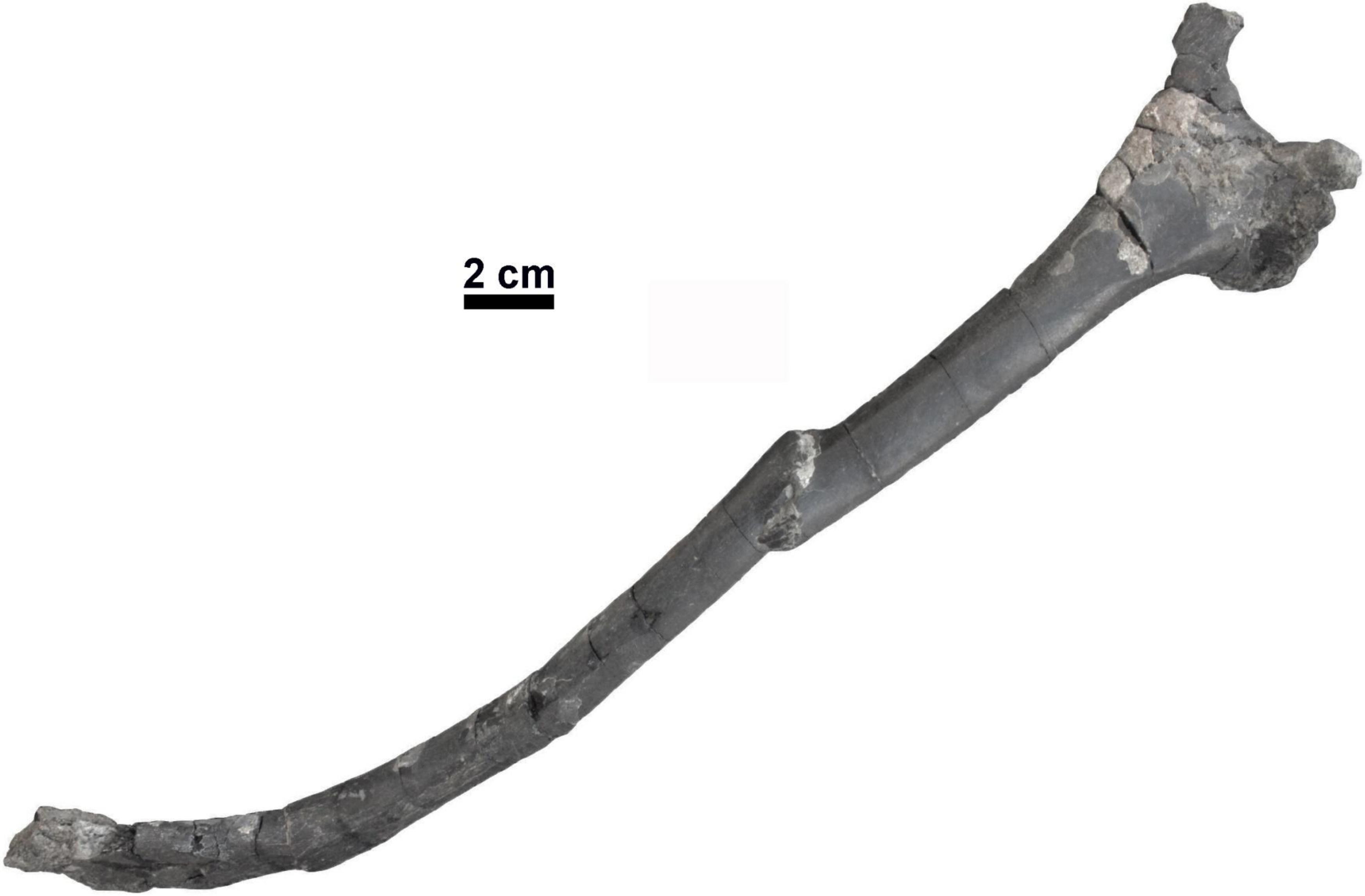

Left ulna, LPB (FGGUB) R.2807, in medial view.

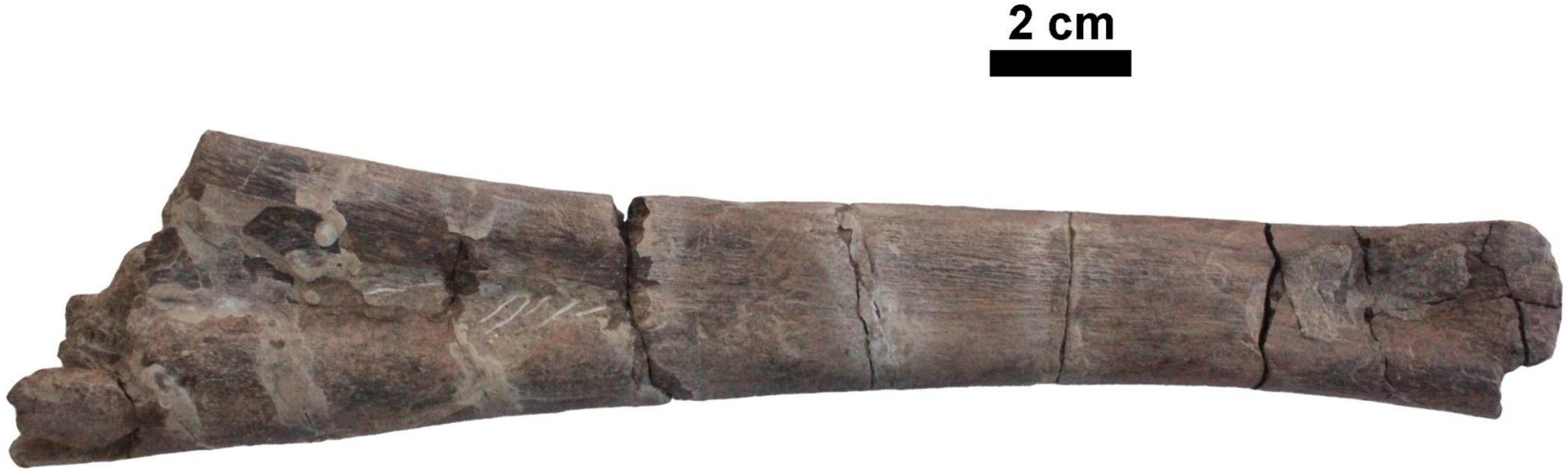

Left tibia, LPB (FGGUB) R.2812 in lateral views.

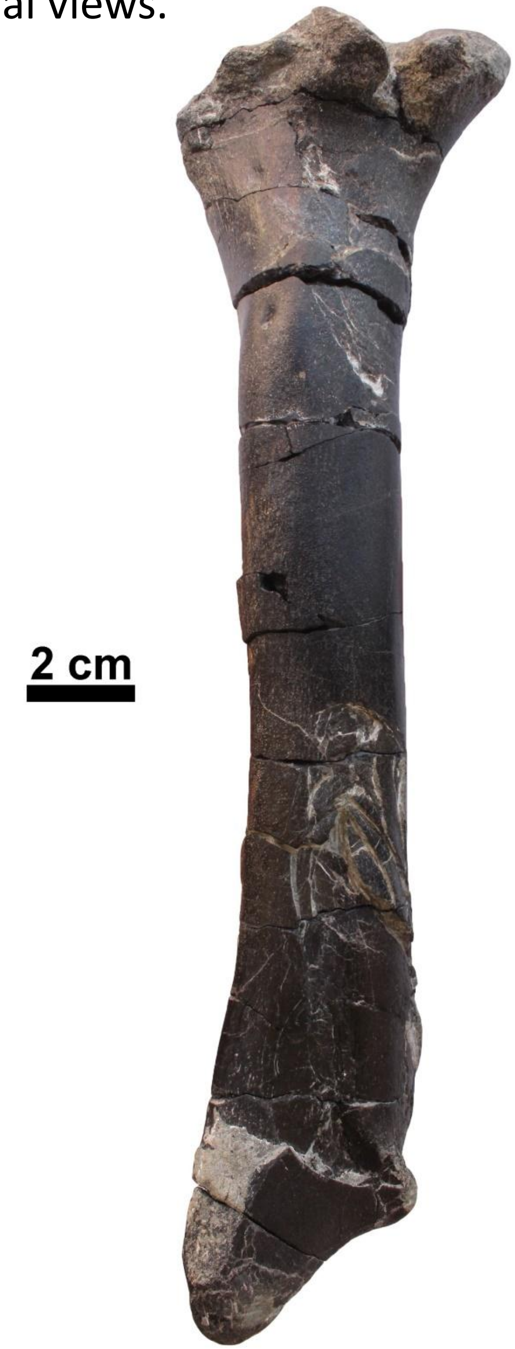

Right fibula, LPB (FGGUB) R.2814, in medial view.

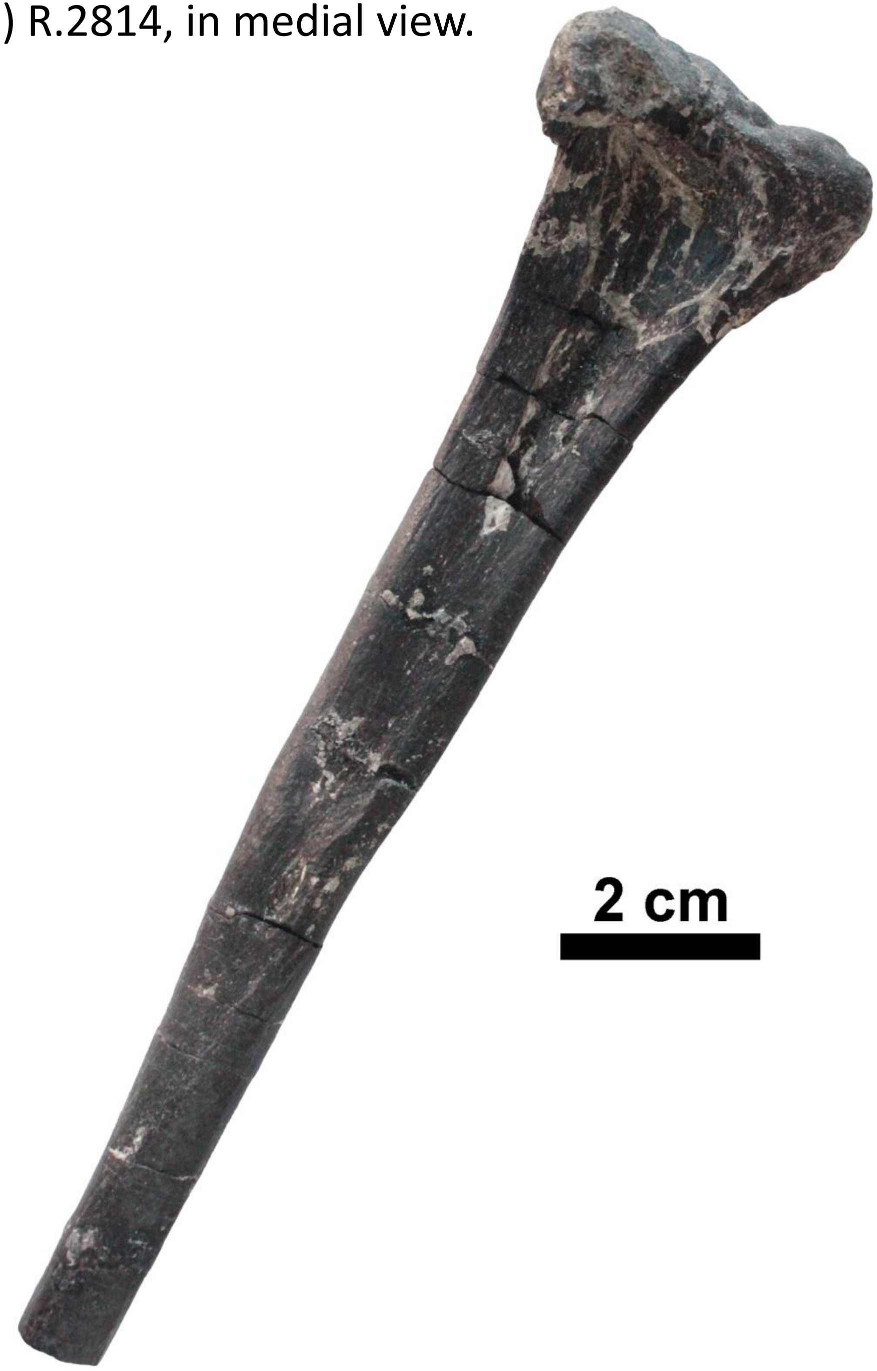

Third metatarsal, LPB (FGGUB) R.2816, in lateral view.

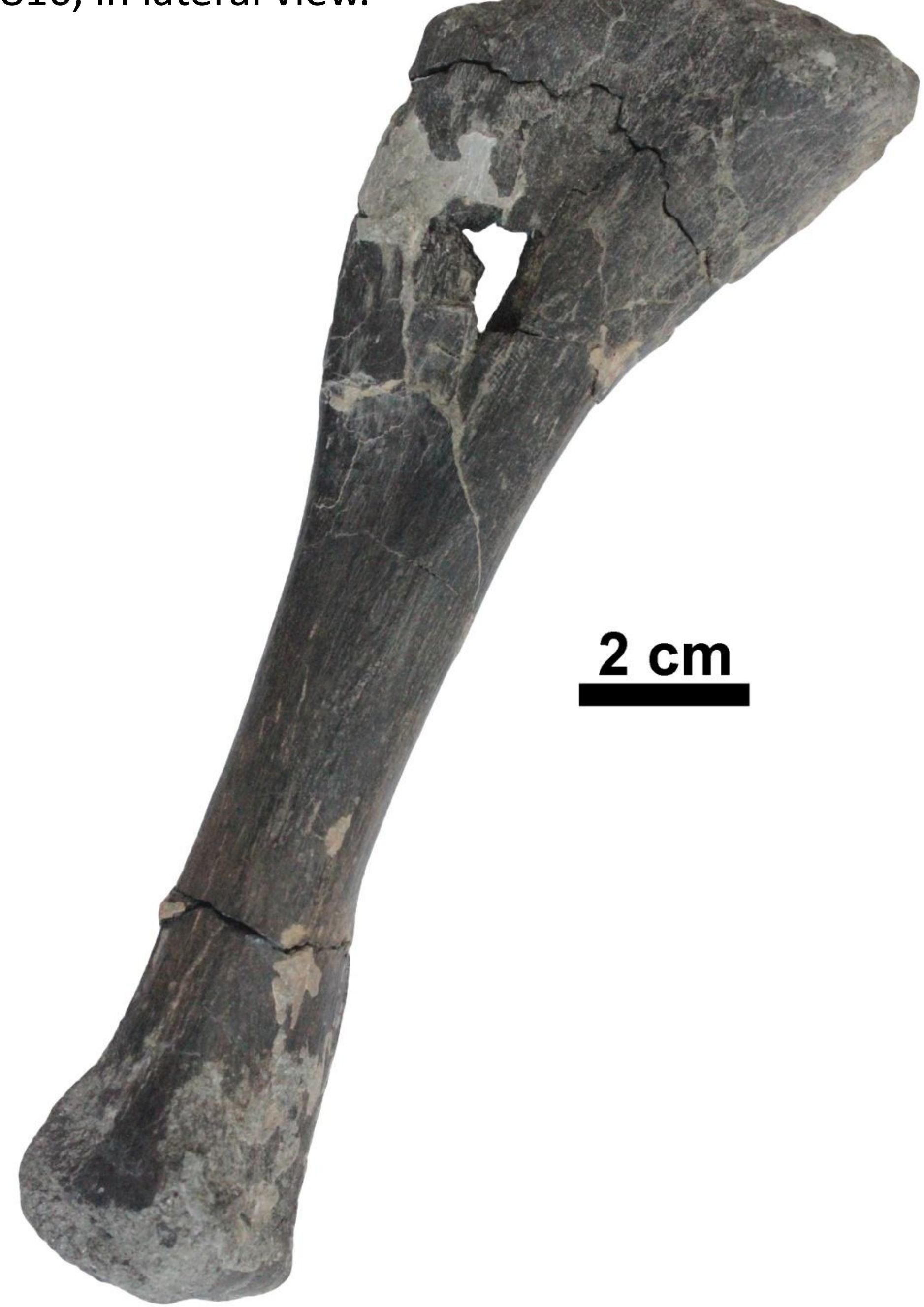

Pedal phalanx, LPB (FGGUB) R.2822, in dorsal view.

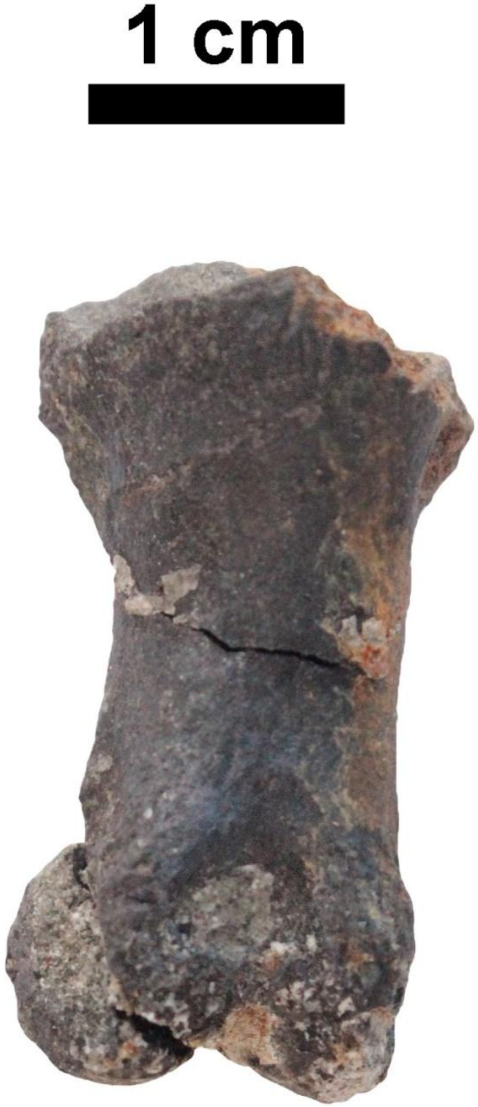

Figure 11: Generalized skeletal reconstruction of the ‘K2 titanosaur’, Vălioara (Densuș–Ciula Formation).

Note:detailed investigation of this material is currently in progress

Dorsal vertebra No. 253, in anterior view.

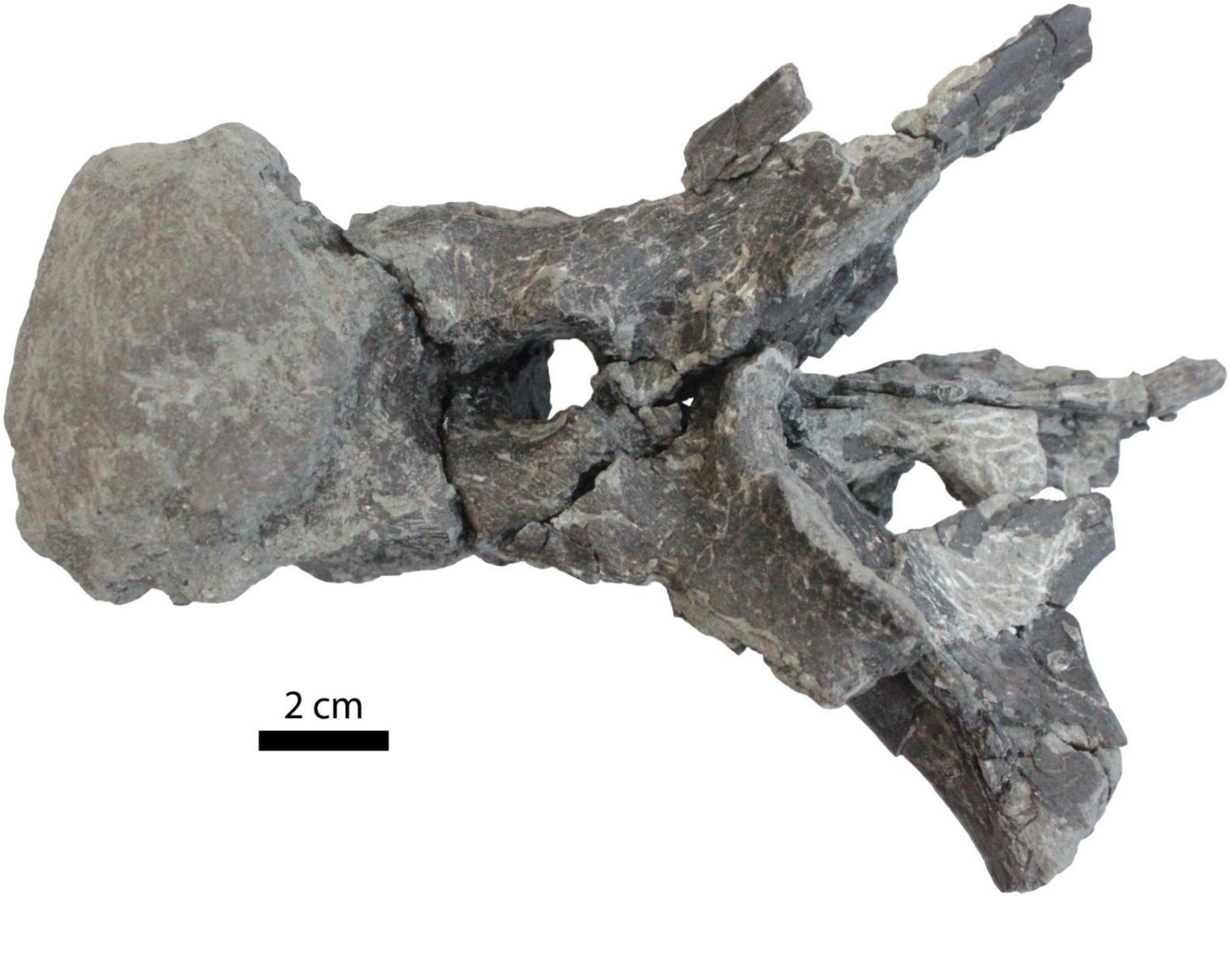

Partial sacral ribs No. 248, in dorsal view.

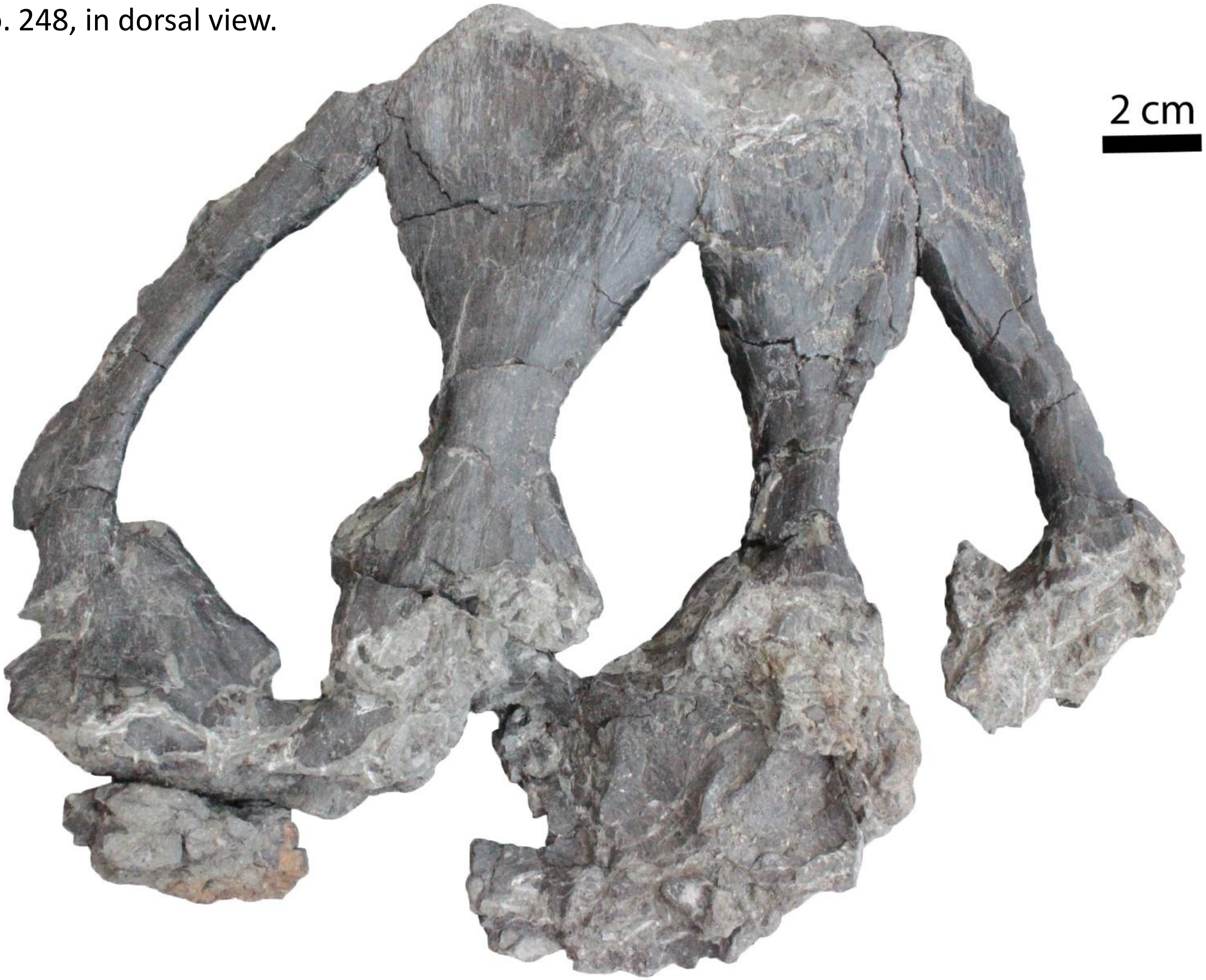

Right? ?pubis fragment No. 195, in lateral? view.

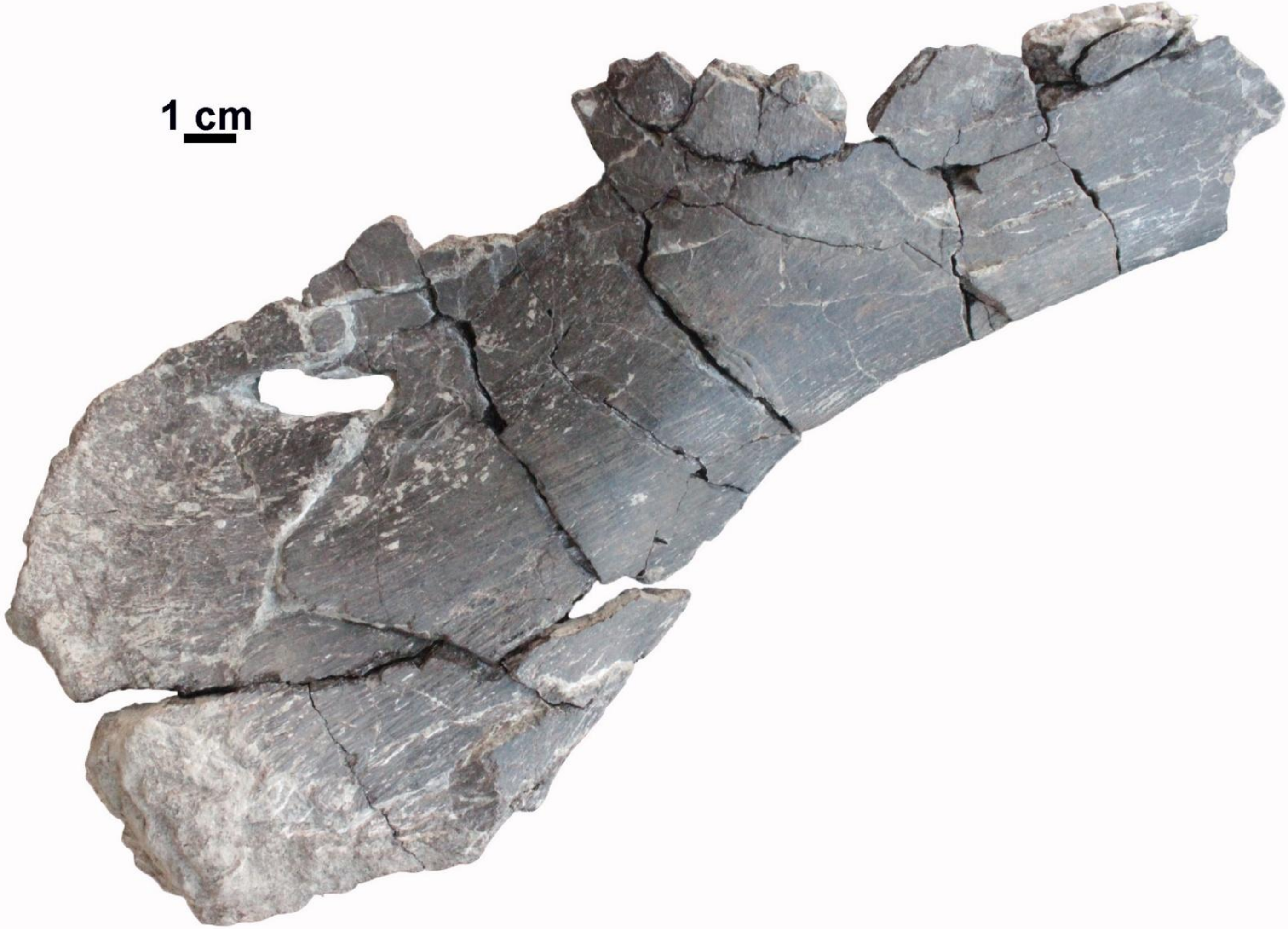

Anterior caudal vertebra LPB (FGGUB) R.2715, in ventral view.

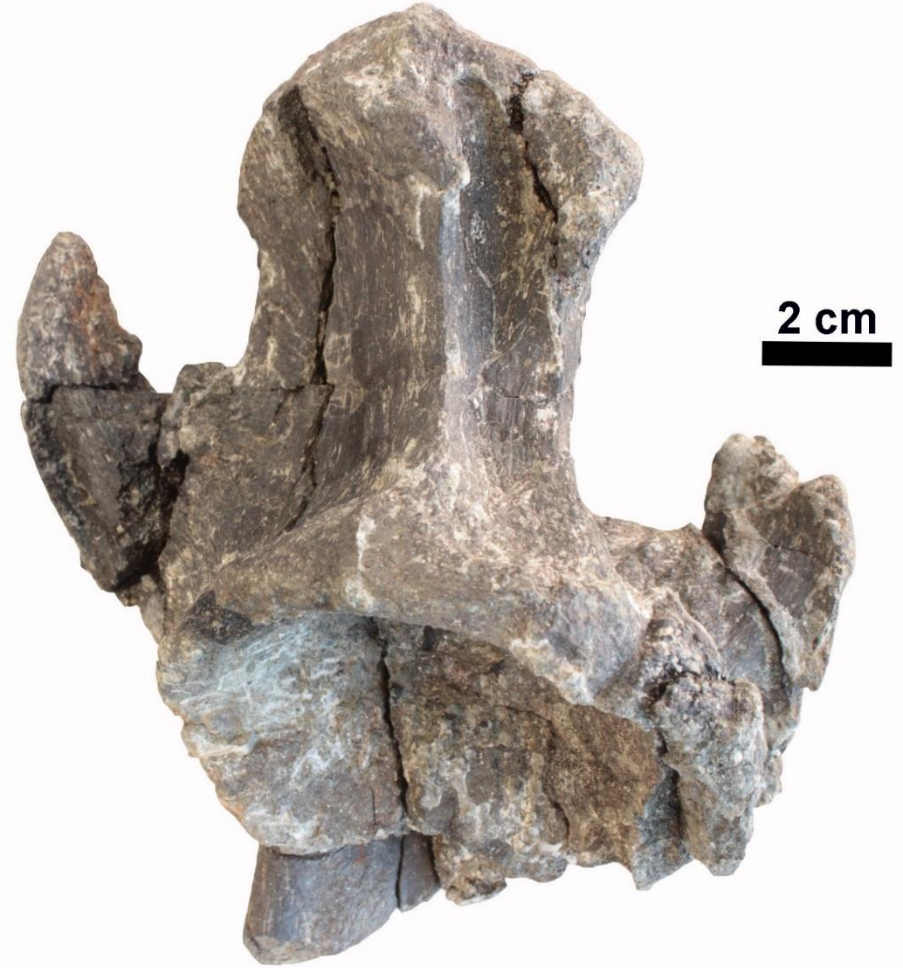

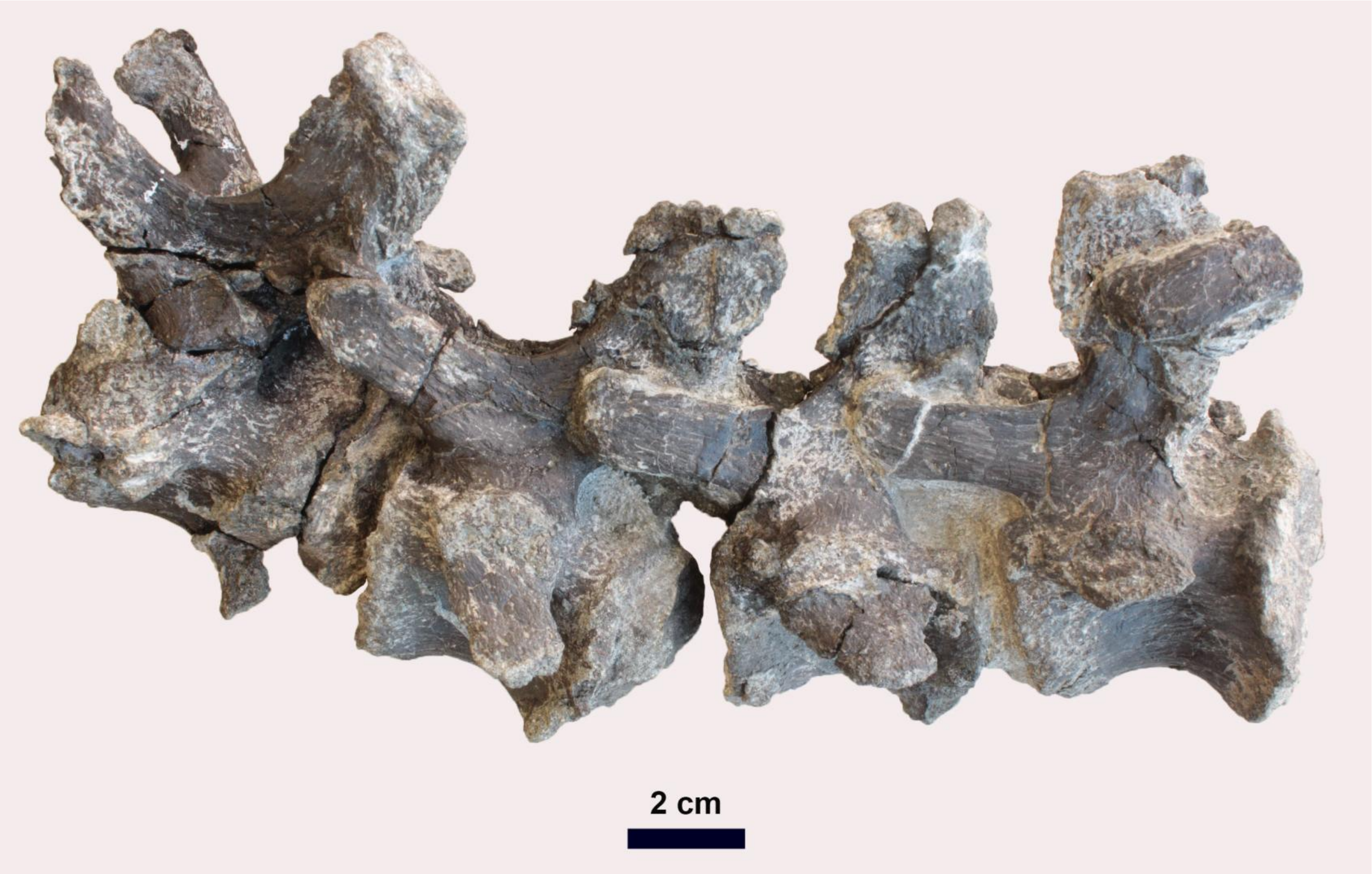

Articulated anterior mid caudal vertebrae LPB (FGGUB) R.2715, in left lateral view.

Posterior caudal vertebra No. 365, in left lateral view.

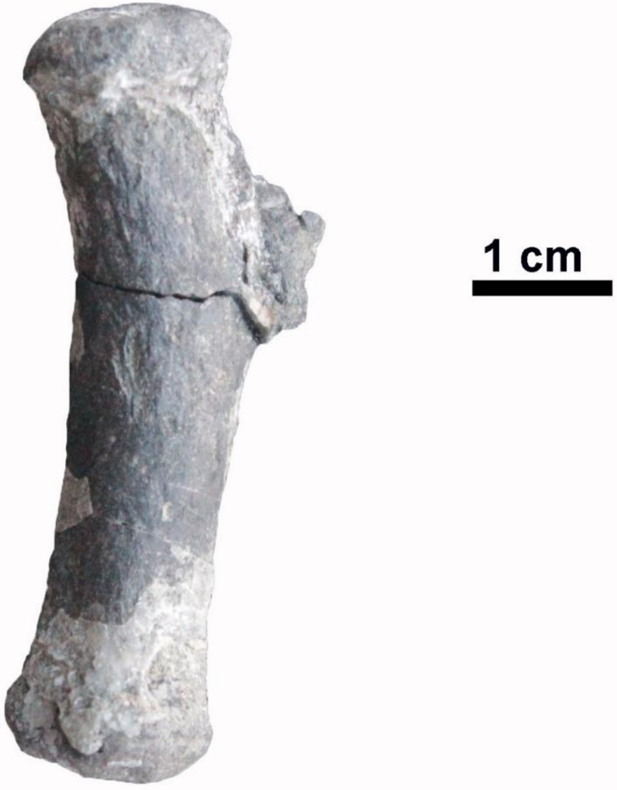

Chevron No. 515, in anterior view.

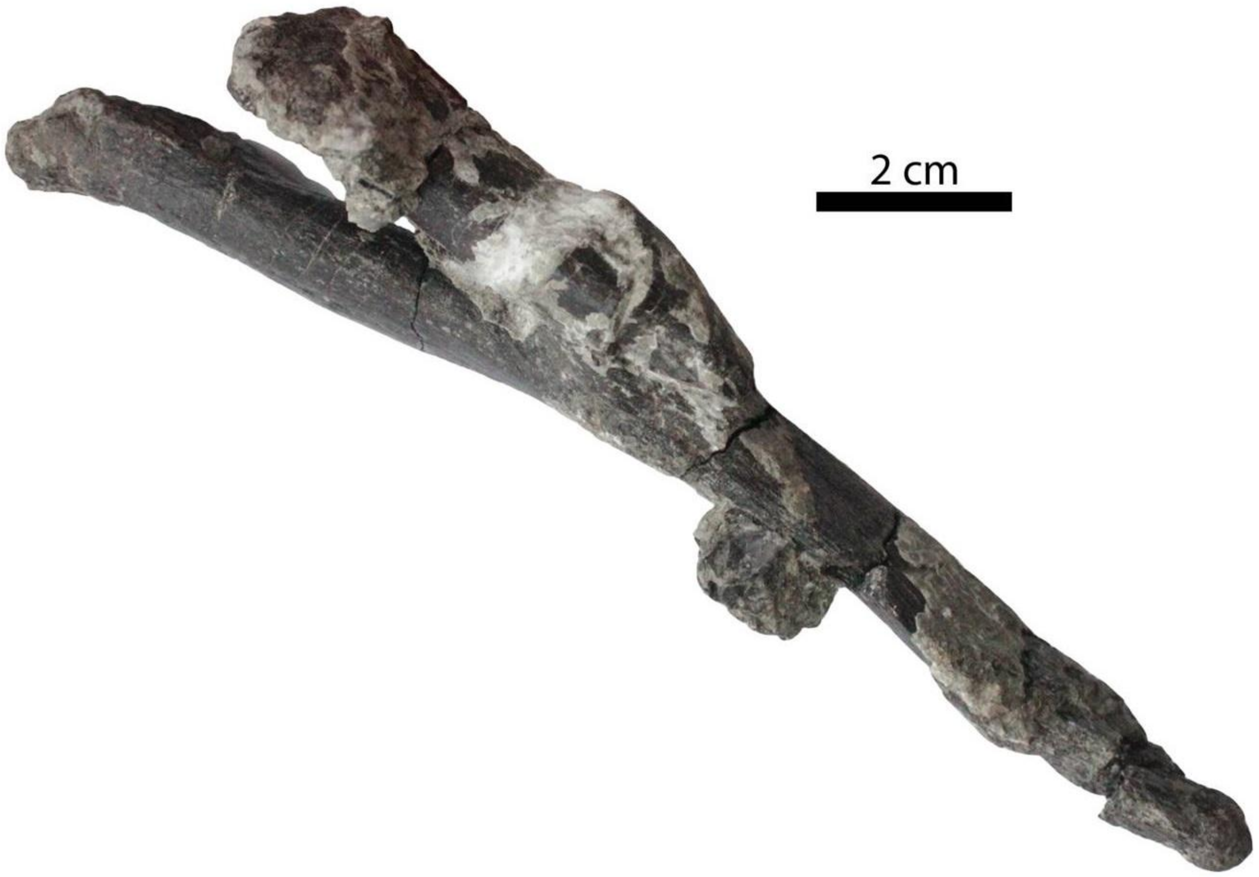

Right humerus fragment No. K2(2022)51, in anterior view.

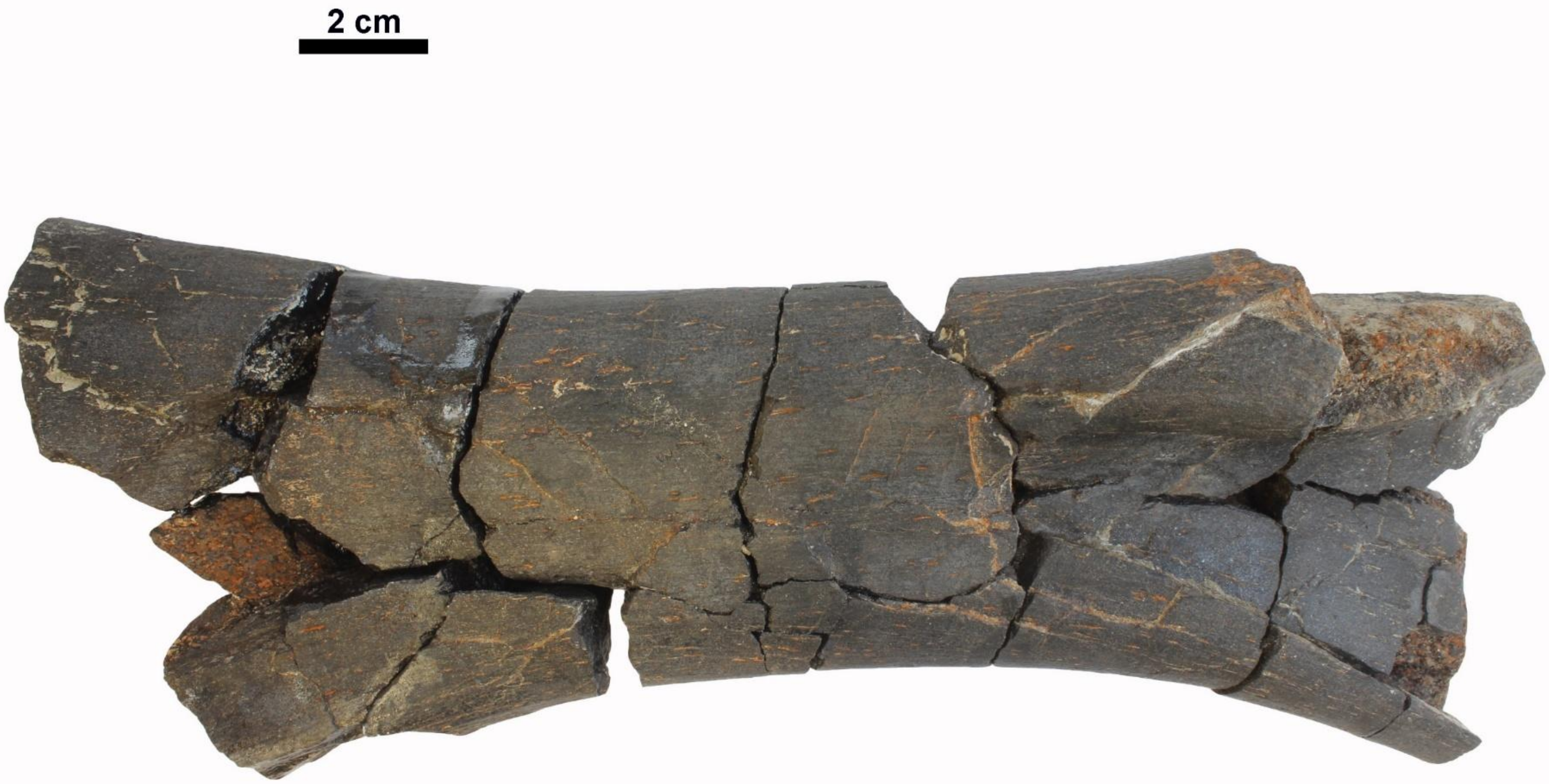

Metapodial element No. 191, in lateral view.

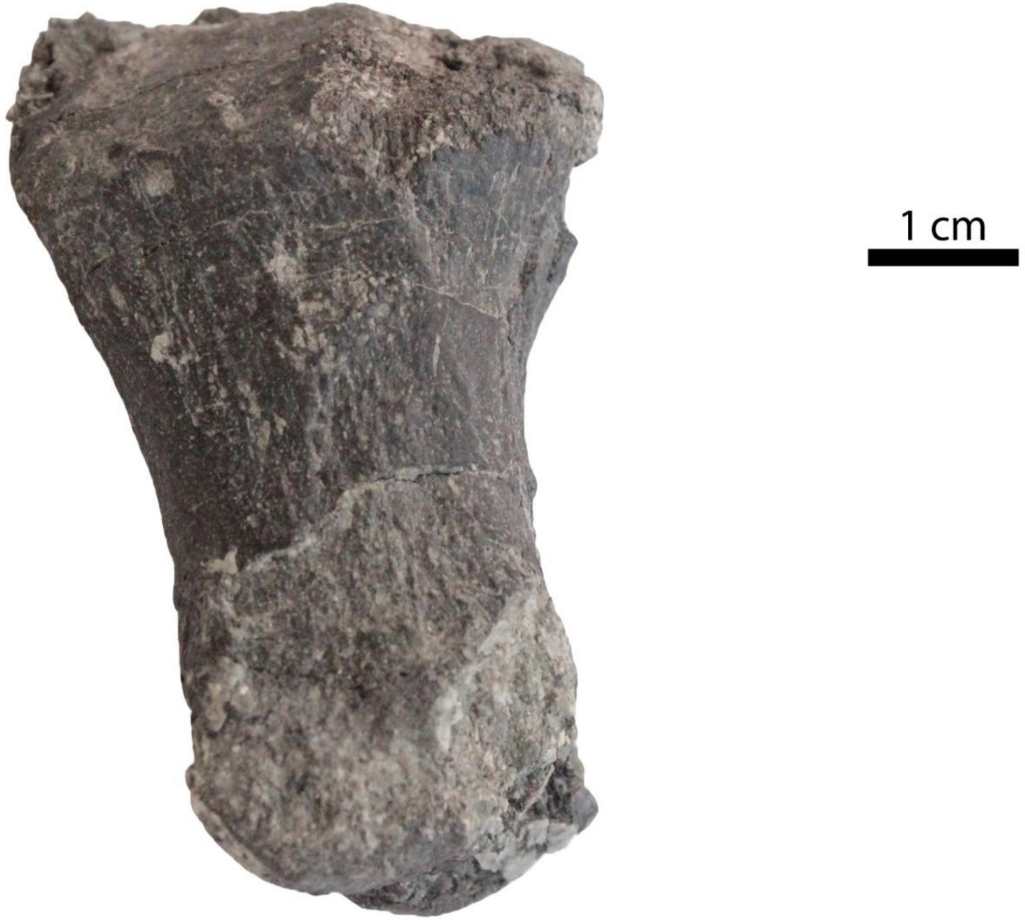

Fragmentary left femur No. K2(2022)53, in posterior view.

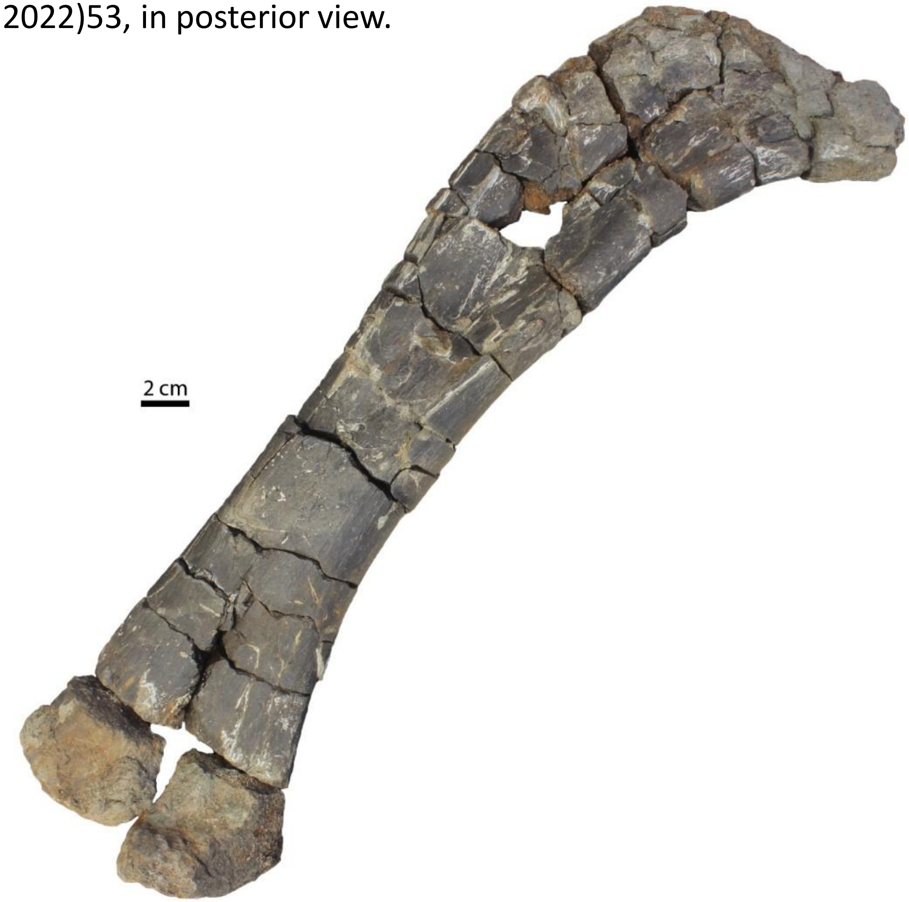

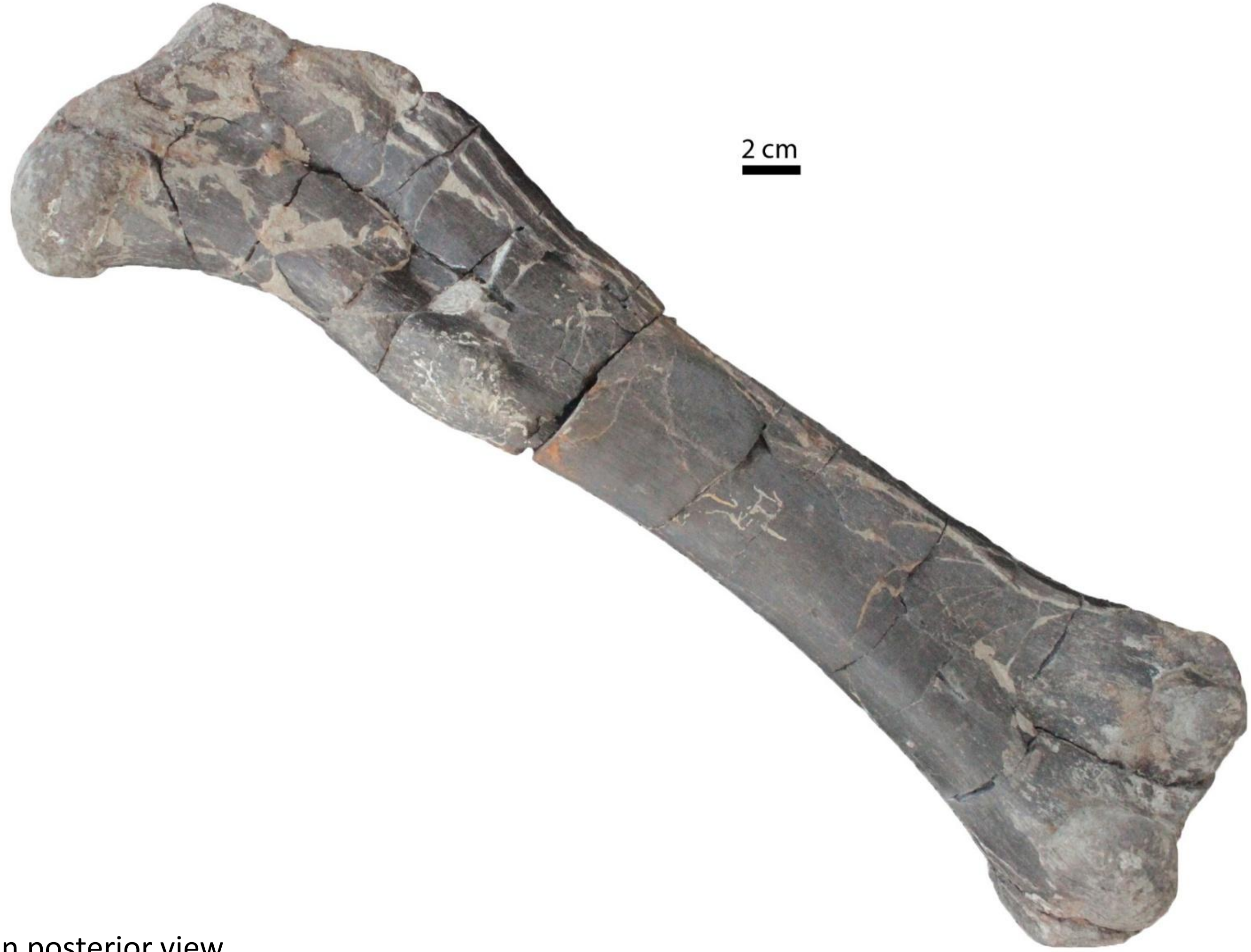

Right femur No. 362, in posterior view.

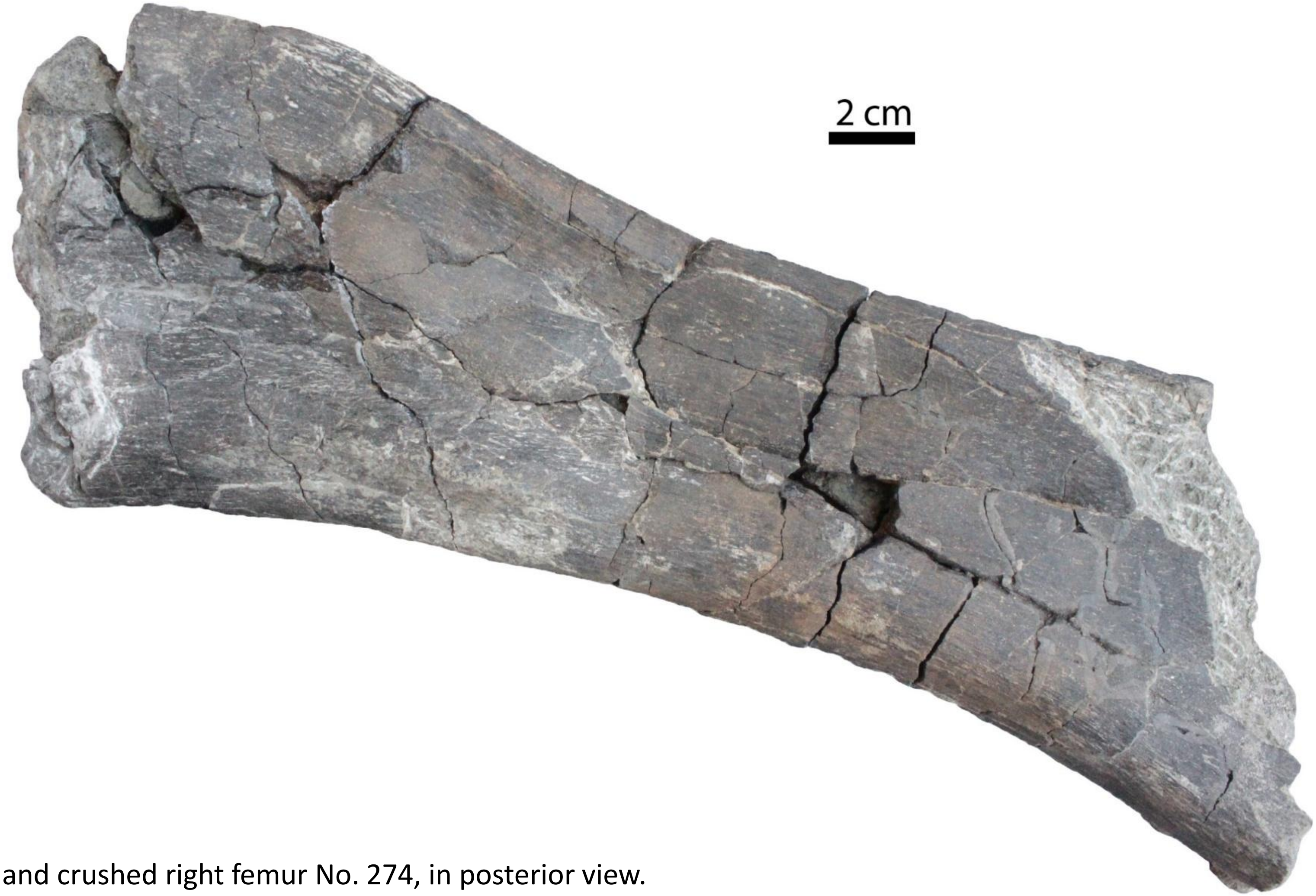

Fragmentary and crushed right femur No. 274, in posterior view.

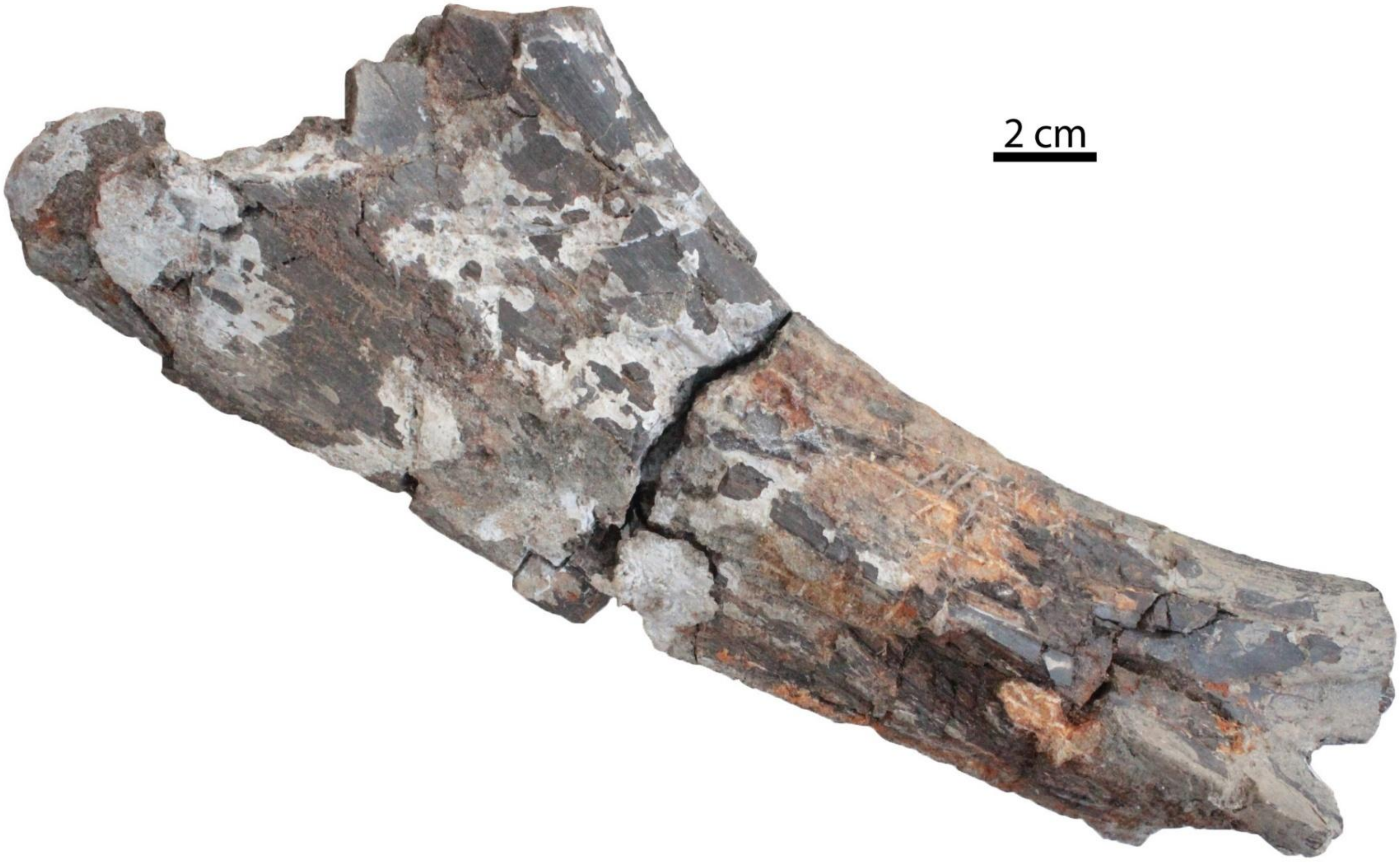

Right? ?tibia fragment No. 317/1, in lateral? view.

Right fibula No. 351, in lateral view.

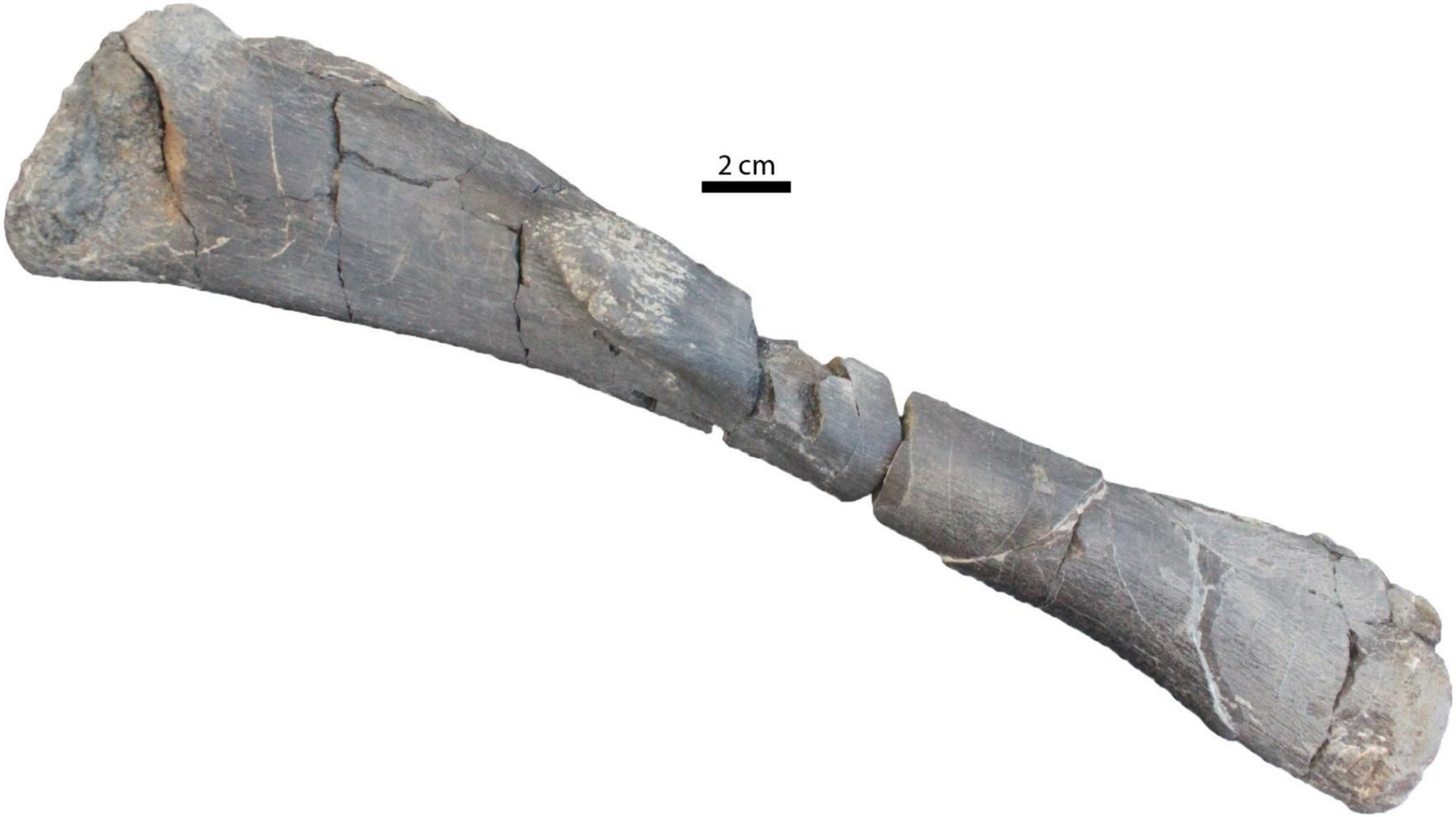

Pedal phalanx No. 313, in dorsal view.

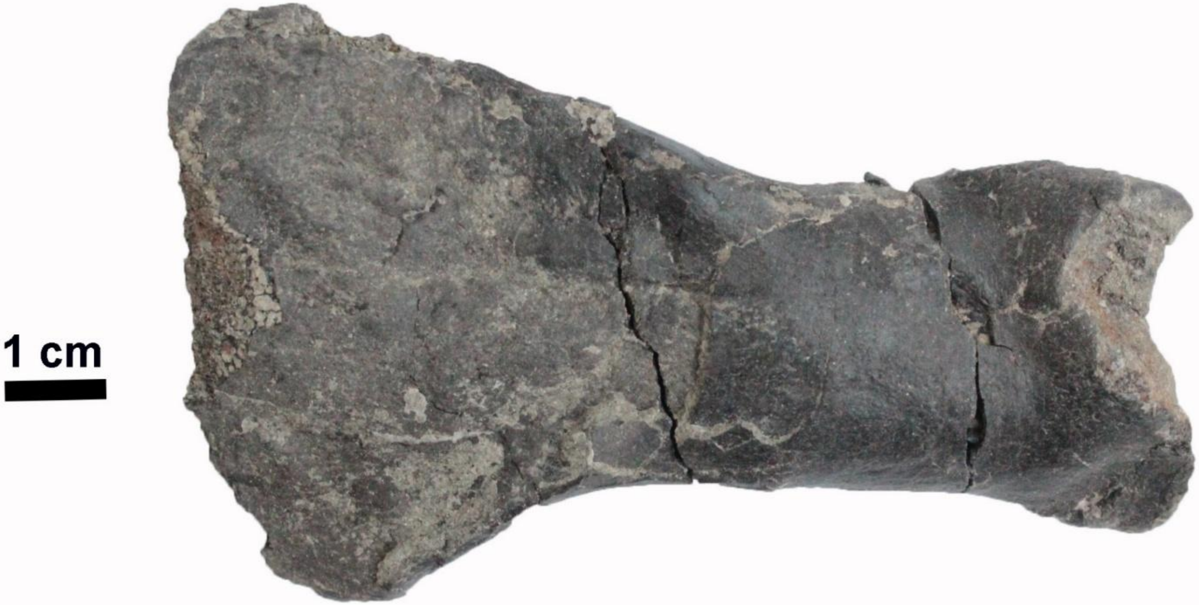

Supplement: S2 File — (PDF) [file pone.0335893.s003.pdf]
