# Supplementary material for: Paleontological and paleoecological significance of the oldest highly productive Upper Cretaceous (lowermost Maastrichtian) bonebed of Haţeg Basin (western Romania; Densuş-Ciula Formation)
Source: PLoS One. 2025 Nov 10;20(11):e0335893. doi: 10.1371/journal.pone.0335893 (PMC12599975; doi:10.1371/journal.pone.0335893)
Supplement: Attachment — Submitted filename: Response to reviewers.pdf [file pone.0335893.s006.pdf]

Response to the reviewers
to the revised manuscript entitled

Paleontological and paleoecological significance of the oldest highly productive Upper Cretaceous (lowermost Maastrichtian) bonebed of Haţeg Basin (western Romania; Densuş-Ciula Formation)

(MS No. PONE-D-25-38937)

by

Gábor Botfalvai*, Zoltán Csiki-Sava, János Magyar, Barna Páll-Gergely, Levente Koczó, Daniel Ţabără, Gergő Konecsni, Soma Budai

Dear Editors and Reviewers

We would like to thank the three reviewers for their constructive and helpful comments and suggestions which have greatly improved our resubmitted manuscript.

Below, we outline our responses to the reviewers' comments, and detail the ways we have modified the original version of the manuscript and supplementary information accordingly. We feel that we have been able to answer all of the comments raised by the reviewers, and hope that our manuscript is now considered suitable for publication in *PLOS ONE*.

Yours sincerely,

The authors

Replies (in black) to the comments of Editor and Reviewers (in red)

Journal Requirements:

In your manuscript, please provide additional information regarding the specimens used in your study. Ensure that you have reported human remain specimen numbers and complete repository information, including museum name and geographic location. If permits were required, please ensure that you have provided details for all permits that were obtained, including the full name of the issuing authority, and add the following statement:

The manuscript contains all necessary information and data related to the fossils mentioned in the study. “All invertebrate and vertebrate remains mentioned in the text are curated as part of the collections of the Laboratory of Paleontology of the Faculty of Geology and Geophysics, University of Bucharest, and are accessioned here under LPB (FGGUB) numbers” (see chapter 3).
We have included the following sentence in the manuscript, which contains all the information regarding permissions: “The described study complied with all relevant regulations and the fieldwork in the Vălioara area had been conducted under permits issued by the Haţeg Country UNESCO Global Geopark and by the National Agency for Natural Protected Areas (REF: 437/STDH/08.04.2022 and 468/STDH/09.04.2024)” (see chapter 3)

We note that the grant information you provided in the ‘Funding Information’ and ‘Financial Disclosure’ sections do not match. When you resubmit, please ensure that you provide the correct grant numbers for the awards you received for your study in the ‘Funding Information’ section.
Please state what role the funders took in the study. If the funders had no role, please state: "The funders had no role in study design, data collection and analysis, decision to publish, or preparation of the manuscript."

We modified the Funding Information as follows:
Financial support for this project was provided by Hungarian Scientific Research Fund and National Research, Development and Innovation Office (Grants no. NKFIH OTKA FK 146097 awarded to G.B.) and a grant of the Romanian Ministry of Research, Innovation and Digitization (Grants no. PN-III-P4-ID-PCE-2020-2570, within PNCDI III awarded to Z.Cs.-S.). The funders had no role in study design, data collection and analysis, decision to publish, or preparation of the manuscript.

Thank you for stating the following in the Acknowledgments Section of your manuscript:
We note that you have provided funding information that is not currently declared in your Funding Statement. However, funding information should not appear in the Acknowledgments section or other areas of your manuscript. We will only publish funding information present in the Funding Statement section of the online submission form.
Please remove any funding-related text from the manuscript and let us know how you would like to update your Funding Statement. Currently, your Funding Statement reads as follows:

We modified the Acknowledgements as follows:
"We are grateful to the 2019-2025 field crews for their assistance in the Vălioara fieldwork, and especially to Attila Ősi (ELTE), Péter Gulyás (Mining Museum in Ajka) and Gábor Falusi (VDRG). We thank the following colleagues for their help provided during this study: Gáspár Albert (ELTE), László Makádi (SZTFH), Marcell Lebán (ELTE), Pál Pelikán† (MÁFI), Cristian Ciobanu (Hațeg Country UNESCO Global Geopark), Maria Raluca Văcărescu (University of Bucharest). This is HUN-REN-MTM-ELTE Paleo contribution No. 429"

We note that Figure 1 in your submission contain [map/satellite] images which may be copyrighted. All PLOS content is published under the Creative Commons Attribution License (CC BY 4.0), which means that the manuscript, images, and Supporting Information files will be freely available online, and any third party is permitted to access, download, copy, distribute, and use these materials in any way, even commercially, with proper attribution. For these reasons, we cannot publish previously copyrighted maps or satellite images created using proprietary data, such as Google software (Google Maps, Street View, and Earth). For more information, see our copyright guidelines: http://journals.plos.org/plosone/s/licenses-and-copyright.We require you to either (1) present written permission from the copyright holder to publish these figures specifically under the CC BY 4.0 license, or (2) remove the figures from your submission:

We have now revised the figure to ensure full compliance with the Creative Commons Attribution 4.0 International (CC BY) license. We have removed all copyrighted material, including data derived from Google Earth imagery, and replaced it with open licensed data (such as Sentinel-2). The updated figure and its caption now rely exclusively on openly licensed and public domain data sources. The updated figure caption has also been modified to explicitly state that the base maps were compiled using these openly licensed sources. We are confident that the figure now meets all of PLOS ONE's licensing requirements and is suitable for publication.

If the reviewer comments include a recommendation to cite specific previously published works, please review and evaluate these publications to determine whether they are relevant and should be cited. There is no requirement to cite these works unless the editor has indicated otherwise.

No such recommendation was made, so this is not relevant to our situation.

Please review your reference list to ensure that it is complete and correct. If you have cited papers that have been retracted, please include the rationale for doing so in the manuscript text, or remove these references and replace them with relevant current references. Any changes to the reference list should be mentioned in the rebuttal letter that accompanies your revised manuscript. If you need to cite a retracted article, indicate the article's retracted status in the References list and also include a citation and full reference for the retraction notice.

We checked the reference list and corrected any further deficiencies we found. We did not cite any articles that had been retracted, so this issue was not relevant to the current work.

Comments of Reviewer 1

The quality of the figures does not fully match the descriptive text; the photos of the fossil are often too small to show the morphological details of the bones described.

In agreement with the reviewer's request, we have increased the size of the fossils shown in the figures (e.g. Figure 8). In addition, we have attached a supplementary file (S3 File) containing larger versions of the photographs of the fossils discussed in detail in the manuscript. We believe that these modifications are sufficient to make all anatomical details observable.

Most fossil vertebrate groups from the site K2 are effectively compared with counterparts from other fossil sites in the basin. However, comparisons for theropods and pterosaurs appear to be lacking; incorporating such information for these taxa would strengthen the study.

As aptly pointed out here by Reviewer 1, in the previous version of the MS we have indeed omitted to address the theropods and pterosaurs in this part of the discussion. In order to address and mitigate this shortcoming, we have now inserted two new sections discussing the significance of these two groups, as well, albeit at very different lengths given the very different taxonomic and distributional information available for them, both locally at site K2 and on a regional level. These new sections were inserted at lines 2178, respectively 2211 of the previous version (see 5.3 subchapter of the revised version), clarifying in some details the relevance and significance of these two groups for local faunal evolution.

I also recommend a revision of the supplemental materials. Some inconsistencies create confusion: Table S2 and Table S3 both list 7 theropod records, but the details provided for these theropod fossils do not perfectly align between the tables or with the main text. Specifically, the main text mentions only 6 theropod elements (3 teeth, 2 limb bones, and 1 vertebra), while the supplementary tables list 7 records, including a theropod fibula that is not mentioned in the main text. Clarifying these discrepancies would improve the supplemental data's reliability.

Thank you for pointing this out, as it was indeed an mistake in the manuscript. We have corrected the data related to theropod material and amended this in the manuscript and all tables (S2 Table and S3 File). The Theropoda material known from the site K2 consists of three teeth, three limb fragments, and one caudal vertebra.

A few minor formatting issues were noted in the manuscript. For instance, a space is missing in line 630, and the content in Table 4 is not correctly aligned.

We have corrected these errors in the revised manuscript.

Comments of Reviewer 2

I recommend that each of the crocodile tooth morphotypes that is described be figured, or a reference be given to another paper in which the morphotype is figured. Also, since the specimens are mostly fairly dark, I recommend considering formatting the figures with a white, rather than black, background. This is not a requirement, just a suggestion to consider.

Following up on the suggestion of Reviewer 2 (but also partly addressing a related one by Reviewer 1; see above) we have decided to split the previous figure 8 that also contained some of the crocodyliform teeth from site K2, into two distinct figures, removing all crocodyliform teeth from former figure 8 and adding a new figure (current figure 9) dedicated exclusively to the diverse corocdyliform tooth sample. This change allowed us both to present larger/more detailed images of other isolated vertebrate remains (current figure 8) and to document graphically all the crocodyliform morphotypes discussed in the manuscript text (current figure 9) – as indicated by the reviewer. We also headed its suggestion concerning the output style of the figures, and used white background to the new Figure 9 (as well).

I only saw one grammatical issue in the manuscript: line 2320: "dominated by far by macrovertebrate remains". I had to read this a couple of times to understand what was being said. I think it would be more effective just to say "dominated by macrovertebrate remains" Also one formatting suggestion: when referring to a figure in another manuscript, eg, lines 406 and 841, use lower case "f" for "fig" so it is clear that it is not a figure in this paper that is being referred to.

Thank you for your comments. We have corrected the problems in the revised manuscript.

Comments of Reviewer 3

I suggest the authors to add “N” and “E” to the latitude and longitude in Figure 1.

We have modified Figure 1 in the modified MS based on the reviewer's suggestions.

Add more details to the stratigraphic column, to make it more clearly and more professional. If possible, please add some outcrop photos to show the contact between the sedimentary rocks and the volcanic units. Please also mark the sample names in A, and mark the fossils and sample names in the column.

We believe that in it is original form the figure was misleading and it led to misinterpretation. Figure panel A shows the stratigraphy of the entire terrestrial unit cropping out in the Vălioara study area, amounting to ca. 1600 m in total thickness based on the work of Albert et al., 2025; while panel B shows the ca. 10 m thick studied interval at the Neagului Valley, near site K2. The figure was modified to show the difference in scale and to indicate the position of the studied section in the more comprehensive terrestrial unit more precisely.
Volcanic/volcaniclastic units are not present neither in the outcrop nor in its vicinity. We believe that the color of regional erosional surfaces in the original figure was misleading as it had a similar color to that used to indicate ‘Pyroclasts’. This detail has now been changed. Furthermore, the figure was complemented with two photos describing the sedimentology of the studied outcrop.

Lines 285-339. Since the authors described the sedimentary facies, representative thin section photos under microscope might be needed here to show the readers more detailed lithological features of the bonebeds.

The nature of the outcrop does not facilitate the creation of thin sections, due to the wetness and friable texture of the studied sediments. The bonebed is thoroughly bioturbated and very heterolithic, and thus we do not think thin sections would reveal any special texture that would add to the original interpretation. In addition, the original bonebed horizon is no longer accessible, so thin section sampling is currently not possible.

Lines 1473-1478. If possible, please provide some radiometric dating evidence on the Densuş-Ciula Formation in Haţeg Basin.

Although we have previously mentioned the existence of such radiometric evidence in the text of the MS (e.g., lines 1949-1953, 1958-1959), this was done indirectly, with reference to previously published studies. In order to address the suggestion of Reviewer 3 in this regard, however, we have decided to introduce at the highlighted part of the MS a brief new section with more details on the relevant, currently existing radiometric data (again, with references to previously published work), emphasizing the geochronological information that supports a 'middle' Campanian age of the beginning of the Densuș-Ciula Formation deposition. This section reads: "Most significantly in this respect, several recently acquired zircon U-Pb radiometric ages derived from primary andesitic-dacitic clasts recovered from volcaniclastic deposits belonging to the Lower Member of the Densuș-Ciula Formation [36] as well as from detritic zircon samples that originate from tuffitic intercalations in the basalmost part of the continental Densuș-Ciula succession [20] fall into the latest early to earliest late Campanian time interval (roughly 81 to 78 Ma), in good accordance with different marine micropaleontological and palynological biostratigraphic constraints currently available from both the continental and the underlying marine beds [11,20,32,35,53]."

According to the description of different degree of abrasion of the fossils, and the relatively quick accumulations of the fossils, it raised me an hypothesis that these fossils might be transported by a pyroclastic flow that deposited within a delta due to the sudden decrease in current energy. Why there was a sudden drop in fluvial transport energy? Were there any volcanic activities nearby to provide the pyroclastic flow? If so, it will be very interesting for better understanding the mass-death event and the taphonomy.

There is no geological data that would indicate a correlation between the K2 deposits and some sort of volcanic activity. The sedimentological profile of the exposed sediments at the site (see Fig. 3B) does not exhibit any indications of pyroclastic material. The youngest (highest-lying) pyroclastic beds are located approximately 500 m lower in the local stratigraphy, thus their occurrence cannot be associated in any way with the bone accumulations observed at site K2 (see Fig. 3A).
According to our interpretation, the energy of fluvial transport suddenly decreased at site K2 site because here the river flowed and discharged into a lake. This marked shift in local hydrodynamics precipitated the rapid accumulation of sediments (and, together with these, that of the transported bones), thereby leading to the formation of a vertebrate fossil-bearing lakeside delta environment.

Some of the discussion should be moved to the supplementary materials, just keep some key points.

It is acknowledged that the discussion in the present article is quite extensive, but it is believed that its contents are necessary in order to address the main objectives of the study, as these were laid out in the introduction. Transferring particular elements of the discussion to various supplementary materials would compromise the comprehensibility of the results, as many of the details discussed in its different parts are intimately interconnected. Nonetheless, the chapter is divided into subsections, thus enabling the reader to skip different parts if and as required.
